# Formulation‐based approaches for dermal delivery of vaccines and therapeutic nucleic acids: Recent advances and future perspectives

**DOI:** 10.1002/btm2.10215

**Published:** 2021-05-04

**Authors:** Marwa A. Sallam, Supriya Prakash, Ninad Kumbhojkar, Charles Wyatt Shields, Samir Mitragotri

**Affiliations:** ^1^ John A. Paulson School of Engineering and Applied Sciences, Wyss Institute of Biologically Inspired Engineering, Harvard University Cambridge Massachusetts USA; ^2^ Department of Chemical & Biological Engineering University of Colorado Boulder Colorado USA; ^3^ Present address: Department of Industrial Pharmacy Faculty of Pharmacy, Alexandria University Egypt

**Keywords:** antigens, dermatological diseases, nanocarriers, nucleic acids, siRNA, transcutaneous vaccination

## Abstract

A growing variety of biological macromolecules are in development for use as active ingredients in topical therapies and vaccines. Dermal delivery of biomacromolecules offers several advantages compared to other delivery methods, including improved targetability, reduced systemic toxicity, and decreased degradation of drugs. However, this route of delivery is hampered by the barrier function of the skin. Recently, a large body of research has been directed toward improving the delivery of macromolecules to the skin, ranging from nucleic acids (NAs) to antigens, using noninvasive means. In this review, we discuss the latest formulation‐based efforts to deliver antigens and NAs for vaccination and treatment of skin diseases. We provide a perspective of their advantages, limitations, and potential for clinical translation. The delivery platforms discussed in this review may provide formulation scientists and clinicians with a better vision of the alternatives for dermal delivery of biomacromolecules, which may facilitate the development of new patient‐friendly prophylactic and therapeutic medicines.

## 1 INTRODUCTION

There is an increasing clinical demand to use biological macromolecules in the clinic for various applications. The global sales of biologics accounted for over 70% of the worldwide revenue from the 10 top‐selling pharmaceutical drugs.^1^ This is of particular importance for managing severe diseases like inflammatory conditions, cancer, and autoimmune diseases as well as controlling infections and dispensing vaccines. Macromolecular therapeutics, including hormones, peptides, cytokines, antibodies, and nucleic acids (NAs), provide higher specificity and potency compared to small molecule drugs.^1^, ^2^ For example, the sales of tumor necrosis factor (TNF)‐α inhibitors reached $40 billion in 2019 due to increased application in dermatology and the expansion of indications.^3^

However, the successful delivery of biomacromolecules has been challenging due to their complex structure and high molecular weight (between 300 and 1,000,000 Da) in addition to stability issues encountered during manufacturing, storage, and administration.^1^, ^4^ Moreover, a short in vivo half‐life, rapid clearance after injection and eventual degradation due to exposure to proteases and peptidases in the body are additional limitations.^5^, ^6^ As a result, this necessitates repeated injections of high doses to maintain therapeutic concentrations, resulting in adverse effects, and reduced patient compliance.^7^, ^8^ Consequently, alternative routes to systemic administration of biomacromolecules are appealing to overcome such limitations and improve patient compliance. While the dermal and transdermal route has been extensively studied for the systemic delivery of hormones such as calcitonin^9^, ^10^ and insulin,^11^, ^12^, ^13^, ^14^ the dermal delivery of macromolecules such as NAs is of particular importance for the treatment of various skin diseases^15^ as well as for prophylactic purposes in vaccines.^16^

**FIGURE 1:**
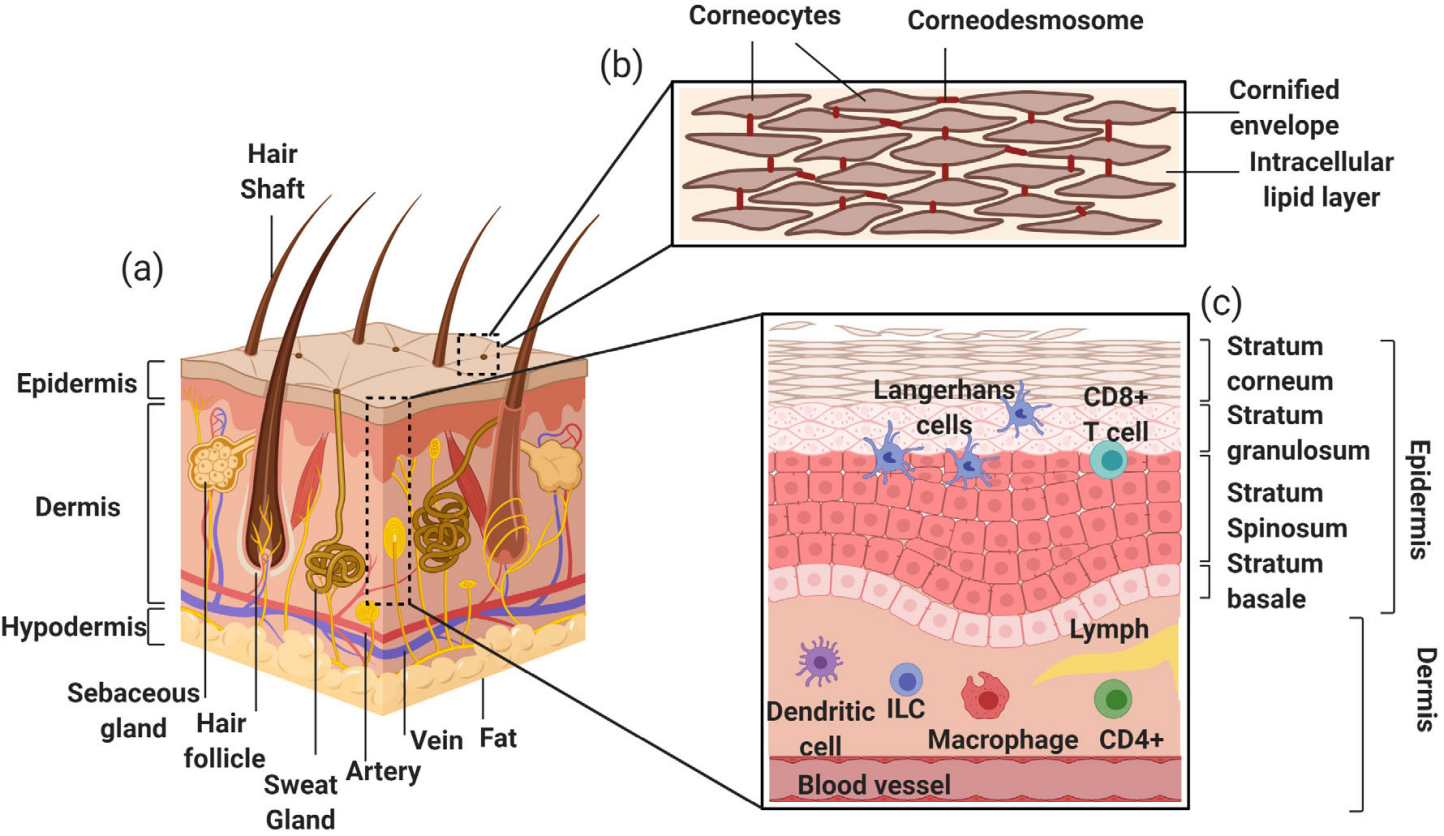
Structure and immunological components of human skin. (a) Illustration of skin structure showing the SC, the epidermis and the dermis, which contains hair follicles, sweat glands and connective tissue, and the hypodermis, composed mainly of adipose tissue. (b) The SC composed of corneocytes connected by corneodesmosomes and is embedded in highly lipophilic layers of ceramides, cholesterol, and fatty acids. (c) Cellular components involved in immunological role of the skin, mainly LCs in the epidermis and DCs in the dermis. (Created with Biorender). DC, dendritic cell; LC, Langerhans cell; SC, stratum corneum

The main biological obstacle for efficient dermal delivery of biomacromolecules is the barrier function of the skin, mainly the stratum corneum (SC). The SC consists of flattened and tightly packed corneocytes embedded in a highly lipophilic matrix composed of ceramides, cholesterol, and fatty acids (Figure 1). This layer is followed by the viable epidermis, which is composed of viable keratinocytes (KCs) and Langerhans cells (LCs). Underneath the epidermis is the dermis, which contains dendritic cells (DCs), lymphatic as well as blood vessels, nerve fibers, collagen and elastic fibers, which gives structural support to the epidermis (Figure 1).^17^, ^18^, ^19^

The barrier properties of the SC limit the penetration of molecules that are less than 500 Da and that have moderate lipophilicity (log *P* = 1–3),^20^ making the dermal delivery of macromolecules such as proteins, antigens, antibodies, cytokines, and NA an exceedingly difficult task.

Physical, or active, methods of penetrating the skin have been studied extensively. This includes iontophoresis,^21^ electroporation,^22^ microneedles,^10^, ^23^ microjets,^24^ and laser ablation.^25^, ^26^ However, despite their efficacy, these techniques suffer from certain limitations including challenges in use over large skin areas. Another important aspect to be considered is to guarantee cargo protection against degradation and to enhance the cellular internalization, when needed, both of which are difficult to control by physical methods. Simpler methods that are passive and noninvasive can facilitate dermal penetration while overcoming the limitations of physical methods. Passive dermal delivery can leverage a variety of tools, including lipid and polymer‐based nanocarriers, peptides, hyaluronic acid (HA)‐derivatives, inorganic nanoparticles, and ionic liquids, among others, for enhanced delivery of macromolecules.

In this review, we focus on the most recent formulation‐based noninvasive passive delivery strategies over the last 5 years for dermal delivery of biomacromolecules, particularly antigens and NAs with applications in transcutaneous vaccination and treatment of inflammatory skin disorders such as psoriasis, atopic dermatitis, cutaneous cancers (e.g., squamous cell carcinoma, melanoma), tissue damage (e.g., burns, wounds), and fibrotic skin conditions.

## 2 NONINVASIVE TRANSCUTANEOUS IMMUNIZATION

### 2.1 Merits of skin as immunization organ

While significant effort is directed toward the development of new vaccines, the most common method for vaccine administration remains to be by subcutaneous or intramuscular injections using hypodermic needles. This makes vaccination painful; some individuals have trypanophobia, and it also requires trained personnel (e.g., intramuscular injections can lead to paralysis in some cases if injected near the sciatic nerve and subcutaneous injections can cause lymphadenitis if injected too deep^27^). Moreover, hazardous medical waste from used needles can be an additional source of infection.^28^ Thus, there is a need for simpler, safer and more effective routes for vaccination.

Noninvasive transcutaneous immunization (TCI) offers a promising alternative to subcutaneous or intramuscular vaccine delivery owing to the large surface area of the skin and its connection to the rest of the body via a network of blood vessels and drain lymph nodes (LNs).^16^, ^29^ The epidermis consists mainly of KCs and LCs. KCs are the main immune effector cell type in skin.^30^ They secrete cytokines, chemokines, and antimicrobial peptides; additionally, KCs express toll‐like receptors (TLRs) that allow the immune system to recognize certain pathogens.^31^ Dermal antigen presenting cells (APCs), including LCs in the epidermis and DCs in the dermis also play an important role in the initiation of immune responses (Figure 1(c)). LCs capture antigens in the tissue environment and present them using major histocompatibility complex molecules. Activated LCs then migrate to present the antigens to CD8+ (cytotoxic) and CD4+ (helper) T cells in the draining LNs. Dermal DCs also play a role in activating the adaptive immune response to induce long lasting protection against those pathogens.^32^, ^33^ Moreover, fibroblasts, which are the major cell component in the dermis, produce cytokines like interleukin (IL)‐6 and transforming growth factor (TGF)‐β to participate in cutaneous immunity.^34^ Accordingly, the skin serves as a highly immunologically active organ that can facilitate a more robust immune response than other tissues that have a limited population of APCs.^35^

In addition to vaccination against infectious diseases, noninvasive TCI is a promising approach for other conditions including allergies,^36^ autoimmune diseases,^37^ and cancer.^38^ The most common food allergy in children is an allergy to cow's milk proteins (CMA). Persistence of CMA is a serious concern due to the risks of accompanying atopic disorders, asthma, anaphylaxis, and other allergen‐related chronic or acute symptoms.^39^ Immunotherapy is considered the only curative treatment method for such allergies.^40^, ^41^ However, conventional oral immunotherapy for CMA requires hospitalization due to the risk of severe allergic reactions, including anaphylaxis. Therefore, a simpler and safer approach such as epicutaneous immunotherapy (ECIT) is required. However, the skin barrier function restricts the penetration of vaccine components such as antigens and NAs. Here we focus on the most recent passive approaches (particularly in the last 5 years) adopted for the noninvasive TCI, and their applications for cancer immunotherapy, protection against infectious diseases and allergy management.

### 2.2 Formulation approaches for noninvasive transcutaneous immunization

#### 2.2.1 Antigen patches

Viaskin® is an epicutaneous delivery system where the powdered antigen is electrosprayed on the surface of the patch. Under occlusive conditions, the antigen becomes solubilized the by natural transepidermal water loss due to emerging perspiration and is passively delivered to the SC. It has been extensively tested as a method for allergen desensitization of sensitized mice in preclinical studies^42^, ^43^, ^44^ and clinical trials for peanut allergy.^45^, ^46^ Tordesillas et al.^47^ showed that topical application of a model antigen with a Viaskin® patch resulted in acquisition of the antigen by epidermal LCs, after which it was transported to the LN, where it was presented to naïve T cells and primed LAP^+^ Foxp3^−^ regulatory T cells (Tregs). In this case, generation of immune tolerance was dependent on uptake through the hair follicle.

A recent placebo‐controlled double‐blind clinical trial used Viaskin® epidermal occlusive patches loaded with lyophilized food antigens for ECIT. This system showed inductions of tolerance to peanut allergies and CMA.^36^, ^48^ However, the efficiency of antigen and adjuvant delivery was low.

#### 2.2.2 Hydrophilic gel patches

In a recent approach to enhance cytotoxic T‐lymphocytes (CTL) production, Kamei et al.^49^ combined a hydrophilic gel patch comprising crosslinked HiPAS™ acrylate medical adhesives, octyldodecyl lactate, glycerin, and sodium HA as a transcutaneous delivery device with mXCL1‐V21C/A59C as an adjuvant. The transcutaneous delivery of ovalbumin (OVA) as a model antigen and mXCL1‐V21C/A59C by the hydrophilic gel resulted in strong induction of OVA‐specific CTLs and inhibited the growth of OVA‐expressing tumors more efficiently than the intradermal injection of OVA with mXCL1‐V21C/A59C.

#### 2.2.3 Solid‐in‐oil systems

In solid‐in‐oil (S/O) systems, the hydrophilic proteins (e.g., vaccine components, allergens) are dispersed in an oil‐vehicle with the assistance of nonionic surfactants. They are prepared by freeze‐drying water‐in‐oil emulsions containing therapeutic cargo followed by dispersion of the surfactant–medicine complexes into an oil vehicle that has affinity to the SC.^50^ This system is attractive as a nanocarrier for peptides such as antigens and vaccine components owing to the high encapsulation efficacy for hydrophilic molecules and capacity for co‐loading immunomodulatory agents like adjuvants.^51^, ^52^, ^53^

Recently, Kitaoka et al.^54^ used a S/O nanodispersion system to deliver both the hydrophilic allergen molecules (β‐lactoglobulin) and the lyophilic adjuvant (R‐848) through the epidermis. The authors applied isopropyl myristate as an oil vehicle in the S/O nanodispersion and sucrose laurate L‐195 as a surfactant.^55^ This system enhanced the penetration of β‐lactoglobulin through pig skin more than fivefold compared to the antigen solution in PBS. The system was then applied as a patch onto intact ears of mice with a model whey allergy. The level of total immunoglobulin E (IgE) was lower, and the levels of β‐lactoglobulin‐specific IgG subclasses were higher compared to similar model mice treated with β‐lactoglobulin in a PBS solution. The extent of ear swelling was lower in mice treated with the S/O nanodispersion, and the cytokines secreted by splenocytes indicated the skewing of the immune reaction toward Th1‐type immunity.

Transcutaneous immunotherapy using S/O nanodispersions has also been implemented for the treatment of Japanese cedar pollinosis, which represents a major health concern in Japan. It is a type I allergic disease treated by subcutaneous or sublingual administration of whole antigens from the pollen extract. Kong and coworkers implemented S/O nanodispersions loaded with vaccine T cell epitope peptides derived from pollen allergen coated with hydrophobic surfactants for TCI in a pollinosis mouse model. This model showed suppression in serum antibody IgE and cytokine production as well as alleviated allergic symptoms compared to mice that received subcutaneous injections.^50^

**FIGURE 2:**
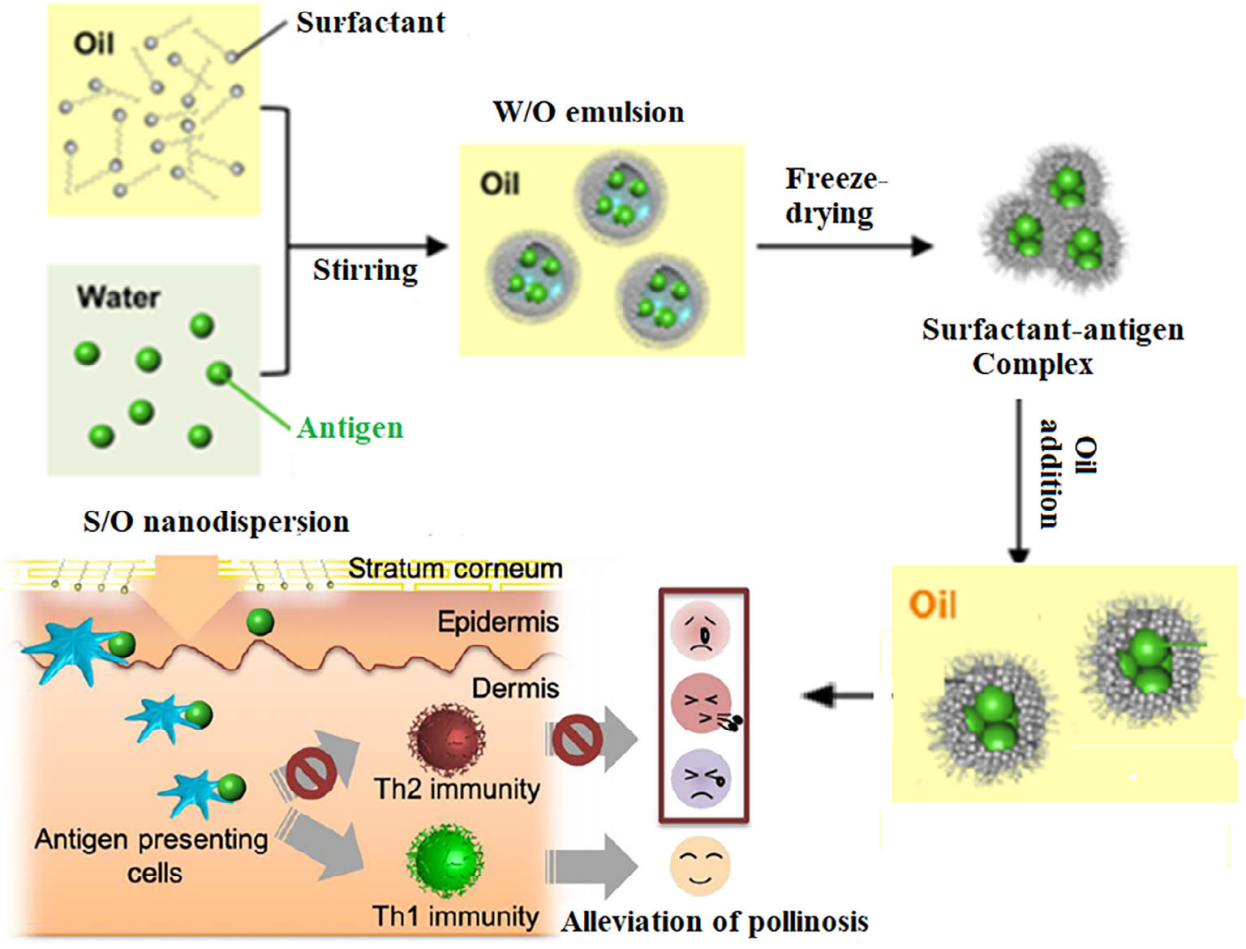
Preparation of a S/O nanodispersion and its application for TCI against pollinosis. Reprinted (adapted) with permission from^56^ Copyright (2019) and^57^ Copyright (2018) American Chemical Society. S/O, solid‐in‐oil; TCI, transcutaneous immunization

In a further advancement, the same group conjugated the pollen extract to galactomannan (PE‐GM) to mask IgE‐binding epitopes in the PE and incorporated it in a S/O nanodispersion (Figure 2). This system efficiently delivered the PE‐GM through skin and improved uptake by DCs. Topical application in a pollinosis mouse model resulted in reduced antibody secretion and alteration of the ratio of type 1 T helper (Th1)/ type 2 T helper (Th2) cells, achieving a comparable therapeutic effect to subcutaneous injections, demonstrating the potential to alleviate Japanese cedar pollinosis.^56^

Recently, transcutaneous immunotherapy against cancer with a S/O system was demonstrated. This system successfully delivered OVA, a model antigen, through the skin and induce OVA‐specific antibodies. Mice vaccinated with OVA‐S/O were challenged with E.G7‐OVA thymoma cells, which express OVA. A significant inhibition of tumor growth was observed in the vaccinated mice.^58^

In a further advancement, Wakabayashi et al.^57^ introduced a transcutaneous vaccine against melanoma by incorporating tyrosine‐related protein 2 peptide, (K‐TRP‐2), as a peptide antigen against melanoma in a S/O nanodispersion coloaded with the adjuvant R‐848. The S/O showed enhanced skin permeability of the peptide. A significant inhibition of melanoma growth was observed in mice vaccinated with this system in addition to the suppression of lung metastasis, highlighting the potential of the S/O nanodispersion as a transcutaneous antigen carrier for cancer vaccines. This technology is simple enough that self‐medication could be possible, making this an attractive system for clinical translation.

#### 2.2.4 Hyaluronic acid conjugates

HA is a linear polysaccharide present in the extracellular matrix of the skin. It is known to promote KC proliferation as well as elasticity regeneration, and it is used in dermatologic clinics as dermal fillers.^59^ HA receptors are highly expressed on skin cells, as epidermal KCs and dermal fibroblasts contribute to antigen recognition by producing immune mediators and presenting antigens to local DCs.^60^ HA is a hydrophilic molecule that also has a lipophilic patch domain, giving it an amphiphilic nature that allows it to diffuse through the SC. It is reported to induce disordering of lipid organization within the SC and cause structural changes in keratin.^61^ HA has been widely implemented as a delivery agent of microscale particles for intranasal delivery of influenza vaccines.^62^ It also acts as a transdermal nanocarrier of macromolecules, such as human growth hormone^63^ as well as small molecules^64^ and peptides.

In the skin, HA is capable of releasing cargo by degrading into small fragments. The low molecular weight fragments are recognized in the body as damage‐associated molecular pattern molecules, which can potentiate an immune response against released antigens by activating Toll‐like receptors 4 (TLR4) and TLR2 and by stimulating the secretion of different cytokines.^65^ Leveraging these properties of HA, Kim and coworkers^66^ conjugated OVA to HA using a site‐specific coupling reaction. The authors investigated the uptake of rhodamine B (RhoB)‐labeled OVA, HA and HA‐OVA conjugates by human epidermal KCs and murine JAWSII DCs. The cellular uptake of HA conjugates was much more efficient than that of OVA‐RhoB. Moreover, HA‐OVA conjugates induced 2.5‐fold more maturated DCs and significantly enhanced cytokine release compared to OVA or HA alone. In vivo transdermal permeation was studied by intravital two‐photon microscopy, which revealed a time‐dependent increase of HA‐OVA‐RhoB conjugates in the dermis, while OVA‐RhoB remained in the SC. No signs of penetration of the HA‐OVA‐RhoB conjugates through the hair follicles were observed, suggesting that the interfollicular delivery was not the route of permeation. Time‐lapse images suggested that the initial primary delivery route of HA‐OVA‐RhoB is associated with natural wrinkles followed by fast diffusion to the dermis. The interaction of HA‐OVA conjugates with DCs in vivo was studied in transgenic mice 2–4 h after topical administration using depth‐resolved two‐photon microscopy.

To confirm the migration of DCs to the draining LNs, where antigens are presented to the naïve T cells to initiate adaptive immune responses, the authors harvested the draining LNs of the back skin 2 days after topical administration. Fluorescence images showed a large number of HA‐OVA‐RhoB conjugates; however, no OVA‐RhoB and HA‐RhoB conjugates were detected in the LNs. The effectiveness of transdermal immunization was tested by applying the HA‐OVA to the back of BALB/c mice and measuring the anti‐OVA IgG antibody titers. HA‐OVA conjugates significantly increased both humoral and mucosal antibodies, with peak levels at 4 weeks. An OVA challenge at week eight elicited strong immune‐recall responses. The minimum OVA dose in HA‐OVA required to induce strong immune responses via the intact skin was 500 μg (25 mg/ kg), compared to 20 μg (1 mg/ kg) for intramuscular immunization. Pretreatment of the skin with non‐ablative fractional laser beams as adjuvant before the application of HA‐OVA elicited an immune response that was comparable to intramuscular injection of HA‐OVA (1 mg/kg) and higher than that produced by intramuscular injection of OVA alone. Moreover, the transdermal immunization induced both systemic (lgG) and mucosal (lgA) immune responses, whereas intramuscular injection did not elicit the mucosal immune response.

HA‐conjugates can be sterilized by filtration and stored in lyophilized powder forms vaccines to be reconstituted before use. The dissolved HA vaccine can be easily applied to the skin as lotion or skin toner or easily incorporated in skin patches facilitating self‐administration, which may of particular use in countries or regions with limited access to healthcare.

#### 2.2.5 Natural polymeric nanocapsules

Polymeric nanocapsules (NCs) are one of the nanoscale carriers that have been widely employed for vaccination. Having a size similar to pathogens, NCs can be efficiently recognized by the immune system, leading to a potent immune response.^30^, ^67^ They can be formulated using a variety of materials, including polypeptides, polyaminoacids, polysaccharides, proteins, and others that can be rationally designed for site‐specific delivery and controlled release of cargo.^68^ Additionally, their core–shell structure permits the encapsulation of adjuvants in the hydrophobic core and incorporation of antigens or hydrophilic molecules in the polymeric shell to be recognized by the immune system.^69^ Moreover, NCs are reported to facilitate transdermal delivery of a variety of molecules. The type of the polymeric shell in NCs can influence complement activation and immune system interactions as well as their in vivo performance.^70^

Chitosan (CS) is a natural polymer that is known to enhance skin penetration besides its effect as an adjuvant.^71^ Bussio et al.^72^ developed NCs for transcutaneous antigen delivery comprising a polymeric corona made of CS and oily core of vitamin E for its immune adjuvant properties.^73^ The positively charged NCs were prepared using a solvent displacement technique and were then incubated with OVA as a model antigen to guide their electrostatic assembly. The association efficiency of OVA was high (75%), maintaining the antigen integrity as evaluated by western blot. The antigen association in the NC shell promoted interaction with the immune system, which was demonstrated by complement activation while not affecting cell viability of macrophages.

Ex vivo studies using porcine skin showed that CS‐NCs enhanced the penetration of OVA by 15 times compared to OVA in solution. This effect was primarily attributed to their nano‐size (100 nm) that allows for a higher penetration, as the antigen diffuses through different routes, including transcellular and intercellular pathways as well as through hair follicles.^74^ Penetration was also aided by the positive superficial charge of CS‐NCs and their ability to open tight junctions.^75^

In another study, HA‐NCs were prepared with an oily core of α‐tocopherol stabilized by benzalkonium chloride and Lipocol® HCO‐40 (LP‐HCO).^76^ Although the electrostatic interaction was successful in associating OVA with CS NCs and antigens such as influenza antigen,^77^ this approach was not applicable to HA‐NCs. Changing the pH of the protein to at least 2 units under its isoelectric point (4.5) resulted in aggregation. However, incubating the antigen with HA‐NCs without changing protein pH (6.9) resulted in 67% OVA association while maintaining the physicochemical properties of the HA‐NCs and preserving the structure and the integrity of the OVA antigen.

HA‐NCs produced immune complement activation over a range of concentrations, a property similar to vaccine adjuvants like aluminum salts. Studying their penetration across pig skin revealed that the association of OVA to HA‐NCs resulted in 22 times higher and 33 times higher penetration and retention compared to OVA in solution, respectively. The authors proposed that the possible mechanisms by which these glycosaminoglycan‐coated NCs overcame the SC to deliver OVA could be mediated by HA receptors widely expressed on KCs and fibroblasts as well as the hydration effect that forms paths for transport of OVA across the SC to aid in antigen retention within the hydrated epidermal layers.^78^ Moreover, the hydrophobic domains of HA, with its eight CH groups, formed a complex with phospholipids and disturbed the SC, enhancing the skin permeability and facilitating OVA absorption across the skin.^79^

#### 2.2.6 Lipid‐based vesicles

Phospholipid vesicles, including liposomes, transfersomes, and ethosomes, have emerged as popular nanocarriers for dermal delivery of encapsulated vaccines owning to their physiochemical and structural resemblance to biological membranes.^80^ They have the advantage of scalable and cost‐effective production as well as not requiring major consideration for safety issue during pre‐clinical or clinical trials.^81^

##### Liposomes

Since induction of protective T cell immunity by cancer vaccines requires both tumor‐associated antigens and adjuvants to be delivered specifically and efficiently to the epidermal LCs, Wamhoff et al.^82^ designed liposomes to target LCs in the skin. LCs uniquely express langerin (CD207), an endocytic C‐type lectin receptor (CLR). The authors functionalized liposomes by conjugating a specific, glycomimetic langerin ligand employing a heparin‐inspired strategy. This facilitated the endocytosis of liposomes by LCs and enabled the specific and efficient targeting of LCs in a physiologically relevant ex vivo human skin model. To explore the modulation of cellular function, the authors applied the liposomal delivery platform to demonstrate the doxorubicin‐mediated clearance of a langerin+ monocyte cell line. The functionalized liposomes resulted in efficient killing of THP‐1 cells at comparable level to those with free doxorubicin, while being nontoxic to LCs. In this case, the liposomes demonstrate superiority to antibody–antigen conjugates, as they allow the coformulation of antigens and adjuvants. The liposome‐targeted delivery to LCs allows the use of lower adjuvant doses, reducing adverse effects while not compromising the CTL immunity. This immune cell targeted delivery approach is of particular importance for the treatment of LC histiocytosis, which is one of the most common pediatric cancers and is characterized by the formation of lesions of the skin, lungs bone marrow, and other organs due to the abnormal proliferation of langerin+ myeloid progenitor cells.^83^

In a subsequent study, the uptake and intracellular routing of liposomes was studied in model cell lines by confocal and live cell imaging as well as by flow cytometry. The liposomes were made from a mixture of 1,2‐distearoyl‐sn‐glycero‐3‐phosphocholine (DSPC), cholesterol and 1,2‐distearoyl‐sn‐glycero‐3‐phosphoethanolamine (DSPE)‐polyethylene glycol (PEG), and they were made to encapsulate protein antigens. The liposomes were internalized into early endosomal compartments and accumulated in late endosomes and lysosomes of primary human LCs followed by a release of the protein antigens. These data further support the applicability of the targeted liposomal particles for protein vaccine applications.^84^

##### Transfersomes

Transfersomes are elastic liposomes containing edge activators (e.g., surfactants) to form ultra‐deformable vesicles. Their ability to squeeze through the intercellular regions of the intact SC gives them greater potential for dermal antigen transport to APCs compared to liposomes.^85^ Hepatitis B surface antigen (HBsAg) plasmid DNA‐cationic complex deformable liposomes were topically applied onto BALB/c mice, and immunity against the antigen was evaluated by measuring serum anti‐HBsAg titers and various cytokines level (i.e., IL‐4, IFN‐γ) compared to intramuscular delivery of naked DNA and pure recombinant HBsAg. The deformable liposome elicited an immune response comparable to other vaccinations, illustrating the potential of transfersomes as DNA vaccine carriers for effective TCI.^86^ Surface functionalization of transfersomes with DC receptor ligands (e.g., mannose) can further enhance the vaccination efficiency by enhancing cellular uptake.^87^

##### Ethosomes

Ethosomes consist of phospholipid bilayers with high ethanol content that increases the lipid fluidization of the vesicles and of the skin lipids by reducing the melting point of polar head groups in the skin.^88^ Zhang et al.^89^ investigated the impact of membrane composition variations of liposomes, transfersomes and ethosomes on the entrapment and penetration of OVA and saponin in the skin. All of the formulations included cholesterol and/or cationic lipid stearylamine. In vivo transdermal immunization efficiency was tested in mice and compared to a positive control group that received subcutaneous injection of OVA with colloidal Al(OH)_3_ as adjuvant. A negative control was also included, which received topical OVA and saponin. Ethosomes showed the highest serum antibody titers, despite having the lowest antigen encapsulation efficiency, suggesting that the high loading of antigen into the formulation is less important compared to the optimal interaction with lipids in the SC. Both elasticity (deformability) and size distribution are key parameters that affect dermal penetration and cellular uptake,^90^ which can be controlled by varying the lipids and ethanol content.

Despite this positive data, there are two challenges for the clinical translation of ethosomes. One is physical instability wherein the vesicles tend to fuse into larger vesicles, leading to breakage and leakage of encapsulated vaccine molecules. The other limitation is the low viscosity of the ethosomal dispersion, which constrains its dermal use. To address this point, Zhang and coworkers ^91^ incorporated OVA‐ethosomes in carbomer hydrogels. The authors discussed the importance of choosing the solvent to dissolve the polymer and concentration necessary to maintain the properties and stability of the antigen‐encapsulating vesicles. In another study, galactosylated chitosan‐modified cationic ethosomes (GCE) encapsulating OVA showed a higher uptake rate by DCs, induced higher level of IL‐2, IL‐6 and intracellular cytokine‐γ and generated superior serum IgG titers in mice compared to unmodified ethosomes. In a recent advancement, GCEs were coated with HA (Eth‐HA‐GC) using a layer‐by‐layer (LbL) assembly method and loaded with OVA to promote bone‐marrow‐derived DC maturation. Loading this into electrospun silk fibroin nanofibrous mats induced a significant increase in serum anti‐OVA‐specific IgG titers and increased the expression of IL‐2, IL‐6 and IFN‐γ by spleen cells. It also inhibited tumor growth in E.G7 tumor challenge by stimulating anti‐OVA immune response, suggesting the potential application of nanofibrous mats for TCI.^92^

##### Archaeosomes

Archaeosomes are modified liposomes prepared with the polar lipids found in Archaea and display superior chemical and colloidal stability.^93^ The polar lipids composition is composed of sn2,3 ether‐linked saturated archaeolipids, imparting resistance to the bilayer against chemical, physical and enzymatic attacks that destroy ordinary phospholipids bilayers. Phagocytic cells can uptake archaeosomes up to 50‐fold greater than conventional liposomes, making them an attractive vaccine carrier.^94^ Archaeosomes contains polar lipids, which cause pronounced capture by phagocytic and immature APCs via receptor‐specialized uptake, resulting in strong CD4+ and CD8+ CTL responses to the encapsulated antigens.^95^, ^96^ Ultra‐deformable archaeosomes (UDA) are able to cross the intact SC and reach the epidermis.

Higa et al.^97^ solubilized the total antigens from *Leishmania braziliensis promastigotes* with sodium cholate (dsLp) and formulated them within UDA and ultra‐deformable liposomes (UDL). In vitro, the UDA was extensively taken up by macrophages and induced rapid cytokine secretion. Topical application on BALB/c mice twice per week on consecutive days for 7 weeks showed that, despite the immunostimulatory effects of dsLp on macrophages in vitro, it raised no measurable in vivo response unless associated to UDL or UDA. The UDA elicited the highest systemic response, comparable to alum. Although both UDL and UDA acted as penetration enhancers, only UDA succeeded as a topical adjuvant due to its high uptake by APCs. Based on these findings, choosing materials with potent immunostimulatory properties is an important consideration when designing a system for passive dermal immunization.

#### 2.2.7 Virus‐like particles

Virus‐like particles (VLPs) are inert, empty capsids of viruses that retain the virus structure without DNA/RNA from the virus itself. VLPs can be engineered to display antigens on their surfaces.^98^ An example is pathogen mimetic reconstituted VLP vaccine developed against the hepatitis B virus (HBV) antigen.^99^ Reconstituted hepatitis B surface antigen vesicles (HBsAg‐REVs) integrated with monophosphoryl lipid A were prepared using a delipidation‐reconstitution method. The natural HBsAg vesicles spontaneously self‐assembled to form immunogenic spherical VLPs that promoted uptake by competent APCs and targeted LN‐residing DCs.^100^ The humoral and cellular immune responses elicited by HBsAg‐REVs via transcutaneous administration were comparable to the marketed intramuscular hepatitis B vaccine formulation. It was reported that the magnitude of immune response increased with the period of antigen contact with skin.

**FIGURE 3:**
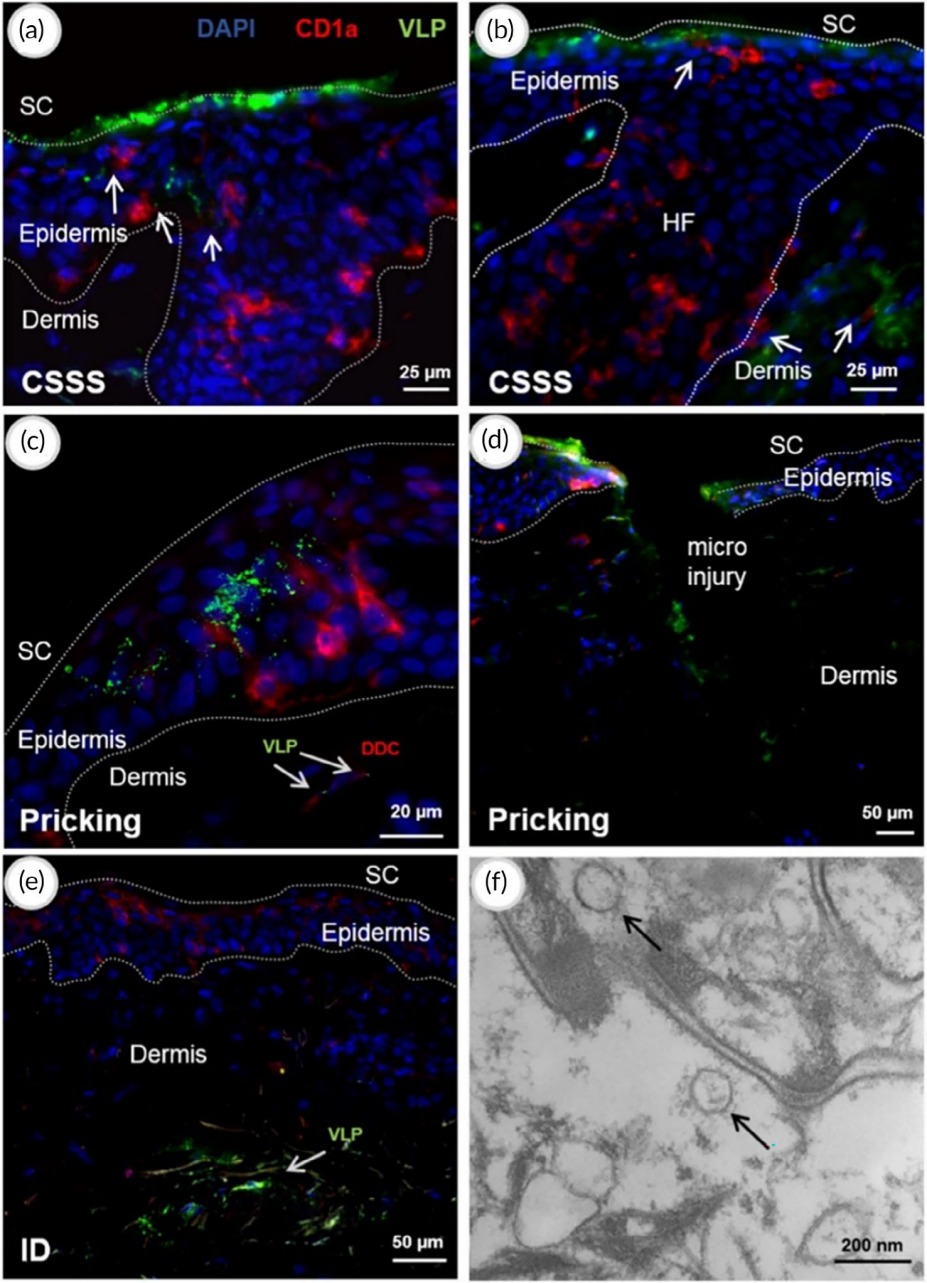
Skin penetration and distribution of VLPs after different administration methods for TCI against HIV. Confocal laser scanning microscopy (CLSM) images after immune histochemical staining with antihuman CD1a antibody (Alexa Fluor®, red). Cell nuclei were stained with DAPI (blue). VLPs were applied on skin after CSSS‐pretreatment (a, b) by pricking with a microneedle (c, d) or by intradermal injection (e). After topical application on CSSS‐treated skin, VLPs (green) are seen on the surface of the SC, near LCs in the epidermis (arrows in [a]) and along the hair follicle canal (arrows in [b]). In sections of skin treated with pricking (c, d), VLPs are found both in epidermal and dermal layers. Arrows show VLPs near CD1a + DCs in the dermis. After intradermal injection, VLPs formed a depot (e). The TEM image (f) shows intact VLPs (arrows) in the SC 16 h after topical administration on CSSS pre‐treated skin. Reprinted (adapted) with permission from^101^ Copyright (2017) Elsevier B.V. CSSS, cyanoacrylate tape stripping; DCs, dendritic cells; VLPs, virus‐like particles

In another study, Runcan et al.^101^ investigated the penetration and cellular uptake of VLPs, composed of the HIV‐1 precursor protein Pr55gag, applied ex vivo to human skin. The authors compared VLP administration on human skin pre‐treated with cyanoacrylate tape stripping (CSSS) to administer by skin pricking and intradermal injection (invasive). CSSS and pricking treatments allowed the penetration of VLPs in the viable skin layers. VLPs were similarly taken up by APCs harvested from culture media of skin explants treated with CSSS and invasive methods. CSSS pre‐treatment resulted in significantly increased levels of IL‐1α in cell culture media compared to untreated and pricked skin. This provided evidence that dermal application with CSSS (mild barrier disruption) is for effective cellular uptake of VLPs that provides stimulatory signals allowing the activation of APCs and uptake of antigenic material (Figure 3).

#### 2.2.8 STAR particles

**FIGURE 4:**
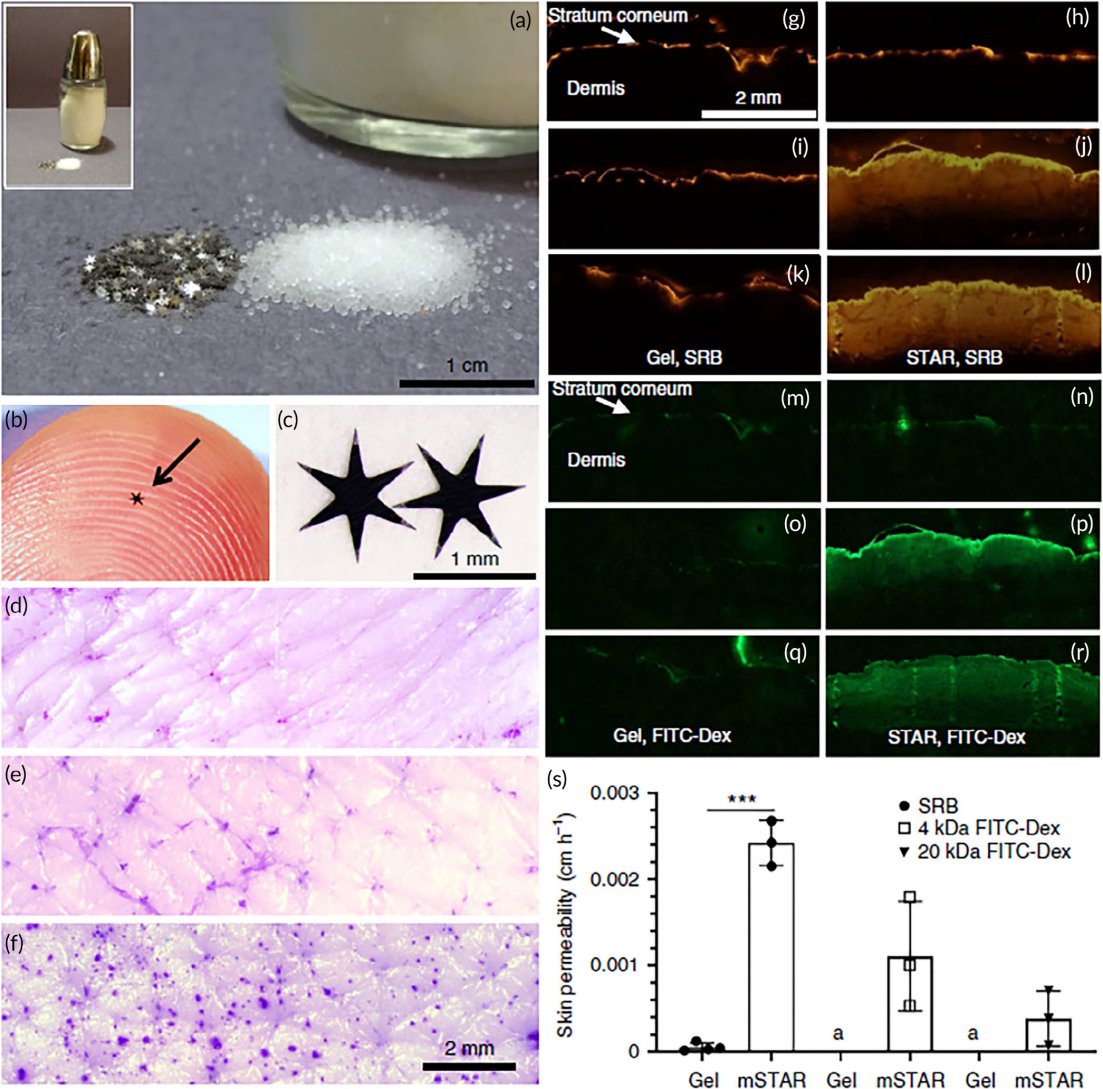
STAR particles. (a) Stainless steel mSTAR (m:metal) particles containing six arms are shown next to table salt for scale; (b, c) mSTAR particles are shown on a fingertip (b, arrow) and on a flat substrate at higher magnification (c). (d, f) Representative micrographs of gentian violet‐stained porcine skin following treatment with *Aloe vera* gel (d), abrasive gel (e), and four‐arm STAR particles. (g–r) Representative histological images of skin sections after delivery of fluorescent molecules to porcine skin treated with mSTAR particles ex vivo, as imaged by fluorescence microscopy. The skin was cryosectioned after exposure to sulforhodamine B (SRB) or 4 kDa FITC‐Dextran (FITC‐Dex) for 1 h (g, h, m, n), 6 h (i, j, o, p), or 24 h (k, l, q, r) following treatment with *Aloe vera* gel (g, i, k, m, o, q) or 5.4 wt% six‐arm mSTAR particles in gel (h, j, l, n, p, r). (s) Skin permeability after treatment with *Aloe vera* gel with or without mSTAR particles and exposed to FITC‐Dex in two different MW in aqueous solution. The “a” mark indicates that Dex was delivered below the detection limit. Reprinted (adapted) with permission from^102^ Copyright 2020, Nature Publishing Group

Star‐shaped, or STAR, particles are millimeter‐scale particles with micron‐scale projections that increase skin permeability and are made of biocompatible materials as aluminum oxide that can be incorporated invisibly into topical formulations similar to conventional skin products. They can be engineered to have different geometries and variable number of arms. Upon rubbing on the skin, their microscopic projections painlessly create micron‐scale pores in the SC, increasing the permeability of topical compounds independent of their physicochemical properties (Figure 4). They provide a simple, low‐cost and well‐tolerated method for increasing drug permeability.

Recently, topical delivery of tetanus toxoid vaccine to mice using STAR particles generated immune responses that were similar to intramuscular vaccine injection. Moreover, application of STAR particles to the skin of human participants indicated they were well‐tolerated and effective in creating skin pores. Star particles could widen the range of compounds that can be topically applied for a variety of skin diseases.^102^

#### 2.2.9 Ionic liquids

**FIGURE 5:**
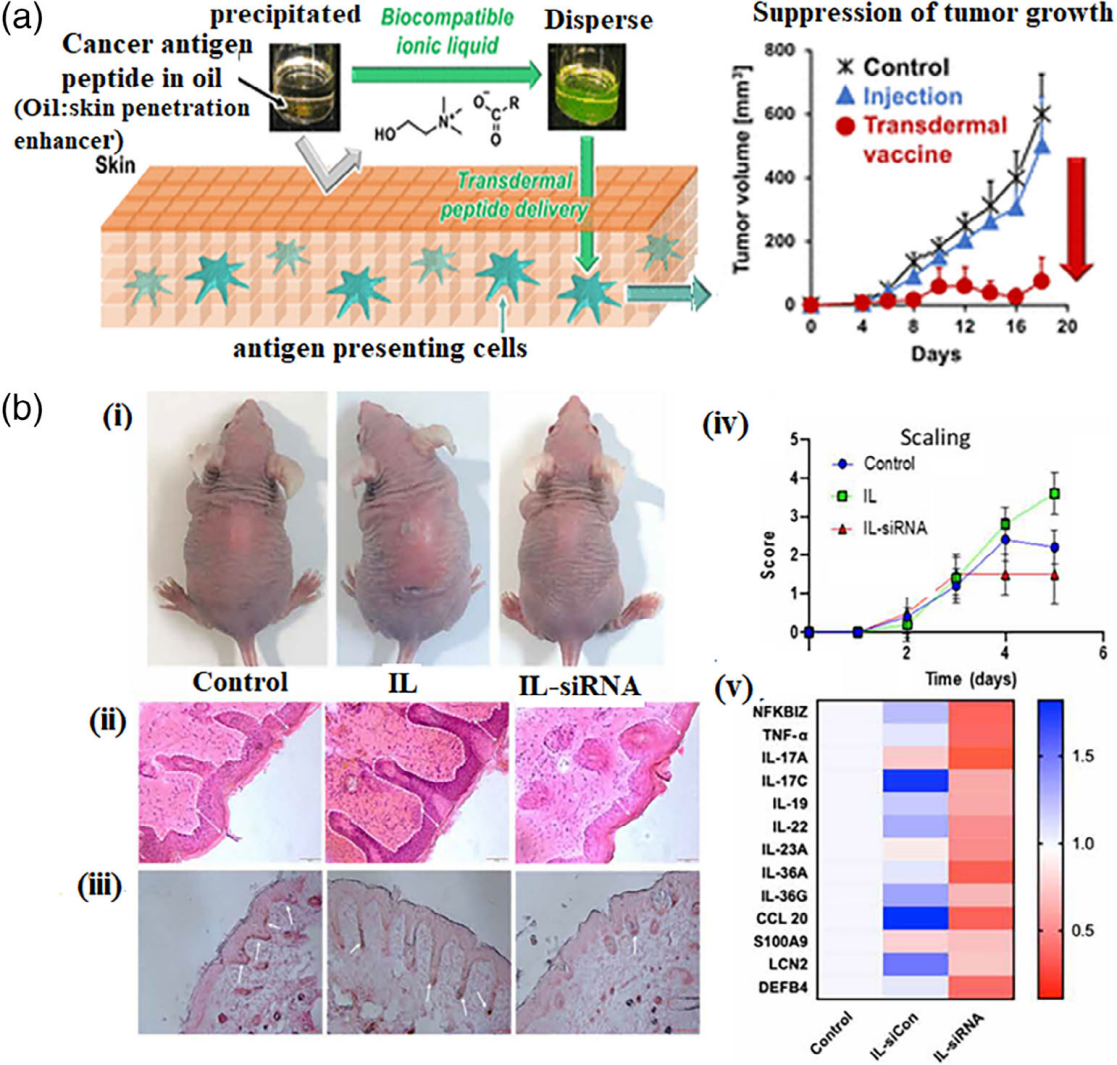
(a) IL‐mediated dissolution of hydrophilic peptides in an oil‐based skin penetration enhancer (right) and suppression of tumor growth in E.G7‐OVA mouse lymphoma model (left). Reprinted (adapted) with permission from ^106^ Copyright (2020). (b) Local inhibition of NFKBIZ by topical IL‐siRNA suppresses imiquimod‐induced psoriasis‐like skin inflammation and other key psoriasis‐associated genes. (i) Psoriasis‐induced mice topically treated with IL‐NFKBIZ siRNA and compared to untreated and IL‐applied groups. (ii) H&E staining of the psoriasis‐induced skin sections from mice with and without treatment. (iii) Immunohistochemistry (IHC) analysis for KC proliferation (Ki67). (iv) scaling scores obtained daily on a scale from 0 (no alteration) to 4 (highly distinct alteration). (v) Heat map for the expression of various psoriasis‐associated genes (h–j). mRNA expression levels were measured by qPCR. Reprinted (adapted) with permission from^103^ under CC 4.0

Ionic liquids (ILs) have gained significant attention for topical and transdermal delivery due to their potential benefits, including a broad safety profile and their ability to tune the physiological properties of active pharmaceutical ingredients.^12^, ^103^, ^104^ Moreover, ILs in vaccine dosage forms can act as stabilizer, solubilizer, targeted delivery inducer, and permeation enhancer.^105^ Tahar et al.^106^ used choline‐fatty acids ([Cho][FA]) as a biocompatible IL to mediate the dissolution of a water‐soluble antigen peptide in an oil‐based skin penetration enhancer. The authors screened a range of fatty acids, among which, oleic acid (C18:1) was found to be the most suitable anion due to its low cytotoxicity, affinity to the oil‐based skin permeation enhancer (isopropyl myristate) and ability to promote the permeation of antigens through skin without irritation in vivo. The flux of transdermal delivery of the peptide increased 28‐fold compared to that obtained delivery using an aqueous vehicle and oil‐based formulations without [Cho][C18:1]. TCI with ILs significantly suppressed tumor growth in an E.G7‐OVA mouse lymphoma model compared to direct injection (Figure 5(a)). The antitumor effect was found to be caused by an increase in CD8+ T cells.

In a recent study, Ukidve et al. synthesized ILs composed of two body metabolites, choline and lactic acid (ChoLa). ChoLa maintained OVA antigen integrity and enhanced immune infiltration at the injection site when administered subcutaneously, leading to a potent immune response against the antigen. This IL was suggested as a promising safe vaccine adjuvant. Moreover, it showed the ability to maintain the stability of SARS‐CoV‐2 spike protein. Although not yet investigated, combining the properties of ILs as skin penetration enhancers with their ability to stabilize antigens may open the door for this class of compounds to serve as bioactive and noninvasive TCI vehicles. More effort should be directed toward the screening of different components for synthesizing ILs that can synergistically enhance immune activity and promote dermal permeation.

## 3 NONINVASIVE DERMAL DELIVERY OF NUCLEIC ACIDS

### 3.1 Potential and limitations of NA delivery to skin

Topical delivery of NAs is emerging as an effective strategy to treat inflammatory skin disorders such as psoriasis,^107^ atopic dermatitis,^108^ cutaneous cancers (e.g., squamous cell carcinoma and melanoma),^109^, ^110^ and skin damage^111^, ^112^ as well as monogenetic skin disorders such as pachyonychia congenita^113^ due to the presence of well‐defined molecular targets that can be silenced to impart therapeutic benefits.^15^, ^114^ The first skin disease to undergo clinical studies using small interfering RNA (siRNA) was pachyonychia congenita, which is an autosomal dominant genodermatosis caused by mutations in keratin. The intradermal injection of siRNA targeting the disease‐relevant mutations showed promising regression of the disease (NCT00716014).^113^ However, frequent intralesional administration was associated with notable pain that hindered its translation efficacy. Consequentially, there is an increasing interest for passive delivery methods that are less painful. Topical administration of NAs is advantageous over systemic administration due to the accessibility of the disease site, avoiding off‐target effects and enzymatic degradation of the NAs.^115^ However, NAs are negatively charged, hydrophilic, large and susceptible to degradation, making their delivery to the skin nontrivial. To address these challenges, a variety of passive delivery systems have been developed in the past few years. In the following sections, we describe these systems and discuss their potential to manage a variety of dermatological disorders.

### 3.2 Inflammatory skin diseases

Atopic dermatitis (AD) is an inflammatory disease that affects 10–20% of children^116^ and 1–3% of adults worldwide.^117^, ^118^ It is associated with itching, severe skin redness, lesions, and papules that significantly have a negative impact on life quality.^119^ Corticosteroids are most commonly prescribed for AD topical treatment; however, their prolonged use has been accompanied by numerous side effects that necessitate shifting to novel therapeutic strategies.^120^ AD is generally associated with the upregulation of inflammatory cytokine transcription factor nuclear factor kappa B (NF‐κB), leading to chronic skin inflammation. NF‐κB is overproduced in macrophages and DCs, and it regulates the expression of different inflammatory cytokines as IL‐6 and TNF‐α.^108^, ^121^, ^122^ Accordingly, the knockdown of NF‐κB with antisense oligodeoxynucleotides showed promising therapeutic outcomes in mouse models of AD.^123^ RelA (p65) is one of the two subunits of NF‐κB along with p50, and it is typically associated with NF‐κB transcriptional activation, which is related to allergy induction. The suppression of RelA by RelA small interfering RNAs (siRNAs) is reported to alleviate the disease symptoms in mice.^124^ Moreover, pathogenesis of AD has been associated with higher levels of some cytokines as IL‐4, IL‐5, IL‐13^118^ and knockdown of these cytokines with topical NA therapies has shown promise in mice.^125^, ^126^

Psoriasis is another inflammatory skin condition characterized by itchy and scaly patches where several genes that encode for proinflammatory cytokines are upregulated in the plaque area.^127^ It is a multifaceted condition involving multiple interactions between the immune system and the KCs. One of the more effective ways to treat psoriatic skin lesions is RNA interference using siRNAs. Once entered into the cytoplasm of the cell, siRNA binds to a specific mRNA and stops further translation of that mRNA transcript.^128^ Therefore, downregulation of the final product is temporary and hence does not lead to any permanent changes.

Several studies have shown the effectiveness of different siRNAs in the treatment of psoriasis in preclinical models. Frequently targeted genes include IL‐23, IL‐17, IL‐36, and TNF‐α, as these genes play a key role in pathogenesis of psoriasis.^129^ Johansen et al. reported the central role of IkBz in the pathogenesis of psoriasis.^130^ IkBz is a transcriptional regulator of several gene products, including IL‐23, IL‐17, IL‐36, and TNF‐α.

Generally, the skin barrier function of the SC is disrupted in psoriasis and AD due to the altered function of ceramide and the sebaceous gland. Accordingly, the major barrier of siRNA delivery to AD‐affected skin are the cell–cell tight junctions (TJs) that control the paracellular pathway in the granular layer of skin.^131^ Hence, an appropriate NA carrier should be able to break through TJs and deliver the siRNA to its target in therapeutically relevant concentrations while preserving its stability.

#### 3.2.1 Peptides

Peptides that promote the transport of NAs into the skin and elicit a therapeutic response have been implemented as carriers for siRNA and gene delivery. TD‐1 is a short synthetic peptide that enabled the successful delivery of anti‐glyceraldehyde‐3‐phosphate‐dehydrogenase (GAPDH) siRNA through non‐follicular rat skin.^132^ Hsu and Mitragotri developed a skin‐penetrating and cell‐entering peptide (SPACE) that was used to deliver IL‐10 and GAPDH siRNA to both mouse and human skin.^133^ SPACE peptide‐mediated delivery to the skin resulted in a knockdown of corresponding gene targets.^134^

Arginine‐rich peptides such as transcriptional activator (TAT) derived peptides have been used to increase siRNA stability against RNase A and promote its transdermal delivery.^124^, ^135^ Topical application of anti‐RelA siRNA (siRelA) and functional peptides in sericin‐based hydrogels showed superior outcomes in reducing the severity of AD in mice model compared to naked siRNA.^136^

In another study, Ibaraki et al.^137^ reported the successful transdermal delivery siRNA using a combination of arginine‐rich peptides composed of arginine cysteine and histidine modified with stearic acid (STR‐CH2R4H2C) and a TJ‐opening peptide (AT1002). Complexation of siRelA with both peptides resulted in higher cellular uptake of siRNA by macrophages and reduced expression of TNF‐α and IL‐6. Moreover, the authors demonstrated that effective siRNA transdermal delivery was accompanied by suppression of the TJ protein ZO‐1 and significant suppression of symptoms associated with AD compared to treatment with naked siRelA.

The use of peptides for noninvasive dermal delivery has also been extended to deliver plasmid DNA. Amphipathic peptide Mgpe9 induced reversible modulation of TJ proteins in the skin, facilitating the successful delivery of plasmid DNA and resulting in efficient gene expression in the basal skin layer without adverse effects.^138^

#### 3.2.2 Lipid nanocarriers

Several recent reports suggest the use of amphiphilic lipid nanocarriers (LNCs) as a vehicle for topical drug delivery of siRNAs. These LNCs, typically positively charged, can interact favorably with the negatively charged siRNA. Their cationic nature also improves cellular uptake and allows endosomal escape of the siRNA. LNCs overcome the significant barrier posed by the SC by temporary fluidization of the lipid layers in the SC, facilitating delivery of NAs into the deeper layers of the skin where they form a reservoir for sustained release.

Lee et al.^139^ reported the efficacy of lipofectamine and cationic dioleoyl‐3‐trimethylammonium propane (DOTAP)‐based nanocarriers for transport of siRNA across porcine skin in vitro. Boakye et al.^140^ delivered a combination of erlotinib (a small molecule drug) and siRNA against IL‐36 using amphiphilic LNCs composed of DSPC, Cetrimonium bromide (CTAB), cholesterol, and pyrrolidinium lipids. The authors reported a marked reduction in the severity of psoriatic plaques in mice with imiquimod‐induced psoriasis after 4 days treatment.

Another LNC, consisting of a glycerol stearate and oleic acid core coated with polyethylenimine (PEI), was reported to deliver a combination of tacrolimus and siRNA against TNF‐α through the skin. This system generated a synergistic effect by reducing the severity of psoriatic plaques in an imiquimod‐induced psoriasis mouse model compared to treatment with tacrolimus alone or TNF‐α siRNA‐loaded nanocarriers alone.^141^

#### 3.2.3 Liposome‐peptide hybrids

The combination of liposomes and functional peptides has been also studied. The incorporation of SPACE peptides in cationic ethosomes was shown to enhance dermal siRNA delivery, resulting in effective gene silencing in vivo. This was achieved either by conjugating the SPACE peptide to the siRNA, conjugating it to the surface of the ethosomal particle or incorporating it directly into the formulation.^142^

In another recent study, liposomes encapsulating siRelA were prepared and designed to have a pH‐sensitive responsiveness. It formed a stable lamella at physiological pH. Under acidic endosomal conditions, the lipid became protonated and resulted in structural changes from the lamellar phase to a reverse hexagonal layer, resulting in collapse of the liposome. To improve the efficacy of the siRNA, AT1002 peptides were incorporated in the formulation to open the TJ of the granular layer by phosphorylating the TJ structural protein ZO‐1, facilitating the penetration of the SiRNA via the paracellular pathway. Studies on RAW264.7 murine macrophages indicated higher cellular uptake and reduced toxicity of siRNA‐encapsulated liposomes compared to naked siRNA. Further, siRelA liposomes with AT1002 showed superior efficacy in alleviating the symptoms of AD and reducing the concentration of inflammatory cytokines in a mouse model.^143^ The effectiveness of this formulation lies in the synergistic combination of AT1002 for its TJ‐opening function and the liposome with its highly flexible structure, allowing for deep dermal penetration of the siRNA.

#### 3.2.4 Lipid‐polymer hybrids

Desai et al. designed a biodegradable, cyclic cationic head lipid‐polymer hybrid NC (CyLiPn) composed of poly(lactic‐co‐glycolic acid) (PLGA) and cationic amphiphiles.^122^ CyLiPn was formed in an aqueous solution by self‐assembly with a negatively charged hydrophobic PLGA core encapsulating capsaicin and a positively charged cationic lipid shell incorporating siRNA against TNF‐α. This system delivered siRNA as deep as 360 μm into skin and showed a significant downregulation of inflammatory genes (i.e., TNF‐α, NF‐κB, IL‐17, IL‐23, and Ki‐67) in a mouse psoriatic model compared to drugs alone, which was comparable to Topgraf®.

#### 3.2.5 Ionic liquids

ILs have emerged as a versatile class of materials for dermal and transdermal delivery of a variety of macromolecules including hormones like insulin,^13^ protein molecules^12^ and NAs.^144^ They are organic salts that are in a liquid state at physiological conditions. ILs have the ability to fluidize cell membranes and extract or displace transport‐limiting lipids in the SC, thereby improving diffusion and facilitating the transport into the SC.^12^, ^145^ Recently, Dharamdasani et al. showed the efficacy of an IL made from choline and geranic acid, termed CAGE, to topically deliver GAPDH siRNA to elicit gene knockdown in the skin.^146^ Building on these results, Mandal et al.^103^ reported the use of a combination of two ILs for topical NFKBIZ siRNA delivery. The authors showed that the choice of IL composition plays a critical role in the stability of the formulation and efficacy of transdermal delivery. CAGE was shown to improve the permeation of siRNA across the skin, whereas choline‐phenylpropionate (CAPA) was shown to improve the stability of the siRNA via favorable stacking interactions between siRNA double helix and aromatic groups in phenyl propionic acid. After 4 days of treatment to mice with imiquimod‐induced psoriasis, the authors showed a significant reduction in the severity of psoriasis plaques, as determined by the psoriasis area and severity index (PASI) score. Significant downregulation of mRNA levels of several psoriasis‐related genes, including IL‐23, IL‐17, IL‐36, and TNF‐α, was achieved (Figure 5(b)).

#### 3.2.6 Liquid crystals

Lyotropic liquid crystalline dispersions have been shown to reversibly reduce the skin barrier properties, facilitating the penetration of siRNA and other macromolecules.^147^, ^148^ Depieri et al.^149^ developed liquid crystalline phase composed of monoolein, oleic acid and PEI that effectively complexed with the IL‐6 siRNA, allowing for improved cellular uptake and resulting in a 3.3‐fold reduction in extracellular IL‐6 in reconstructed human psoriatic skin models while reducing the irritation potential.

#### 3.2.7 Spherical nucleic acids

Spherical nucleic acids (SNAs) are three‐dimensional nanocomplexes of ordered oligonucleotides on a spherical core nanoparticle. SNAs prepared with lipid‐based nanoparticles such as liposomes possess the ability to overcome the barriers to transdermal drug delivery and silence gene expression in the epidermis. Liu et al. reported the use of SNAs to silence the expression of IL‐17 receptor A (IL‐17RA) in mice with imiquimod‐induced psoriasis.^150^ The authors reported a significant alleviation of the psoriatic lesions 5 days after treatment with of IL‐17RA SNA compared to controls.

Nemati et al.^151^ utilized thiolated siRNAs to guide their self‐assembly on the surface of gold nanoparticles. The authors demonstrated improved uptake and transfection of A431 cells in vitro in absence of any specific transfection agents. Using such constructs formulated with Aquaphor® ointment, the authors showed the efficacy of topically delivered EGFR siRNA in vivo in mice with imiquimod‐induced psoriasis.

#### 3.2.8 Dendrimers

Polyamidoamine (PAMAM) dendrimers possess an amine‐terminated surface that can be easily functionalized, providing a positive charge for interaction with siRNA.^152^ Pandi et al.^153^ synthesized cationic PAMAM dendriplex (termed P‐G3 dendriplex), into which siRNA was loaded by simple incubation. The authors compared the therapeutic efficacy of the dendriplex with that of lipoplex (i.e., cationic liposome‐NA complexes) prepared from DOTAP and cholesterol by utilizing them as carriers of TNF‐α siRNA for topical application on a mouse psoriatic model. Both treatments showed significant alleviation of psoriatic skin lesions with no marked differences in the amount of proinflammatory cytokines in the skin, making dendrimers a viable choice for the transdermal delivery of siRNA.

While siRNA remains the focus of the majority of topical NA‐based therapies for psoriasis in recent years, other types of NAs are being studied for similar purposes as well. Examples of such NAs include aptamers and self‐amplifying RNAs (saRNAs).

Aptamers are oligonucleotide‐based macromolecules that can bind to specific epitopes on cellular targets such as proteins. Lenn et al.^154^ designed a highly specific IL‐23 aptamer and demonstrated its permeation through freshly excised human abdominal skin. The aptamer was formulated with an oil‐in‐water cream vehicle. In vitro efficacy studies led to inhibition of Th‐17‐derived cytokines, which have been significantly implicated in the pathogenesis of psoriasis. This was the first report that demonstrated the transdermal delivery of aptamers. Successful transdermal delivery of aptamers may open a variety of cellular targets to be exploited for alleviating symptoms associated with psoriasis and other skin disorders.

saRNAs are derived from the alphavirus genome that encode for alphaviral replicase and the gene of interest. Alphaviral replicase enables the amplification of the gene of interest in the cytosolic region of the host cells, which leads to increased and prolonged in the expression of the proteins. Blakney et al. demonstrated the use of mannosylated PEI copolymers as vehicles for enhanced uptake of intradermally delivered saRNAs in human skin explants.^155^ The authors discovered that higher degrees of mannosylation and higher polymer ratios in the polymer‐saRNA complexes led to higher transfection rates. Saviano et al. reported the efficacy of ornithine‐derived dendrimers for a similar application.^110^ When compared to PEI, the dendrimers showed a ~2–3‐fold improvement in transfection of cells in ex vivo skin explants. These strategies, although not proven in vivo, possess excellent potential for future translation given the ease of formulation.

### 3.3 Skin fibrosis

Skin fibrosis such as hypertrophic scars and keloids affect millions of people worldwide. Fibrotic tissue is like a scar showing a bumpy, irregular and thick surface due to the excessive accumulation of protein under the skin.^156^, ^157^, ^158^ A major cause of fibrotic diseases is the uncontrolled overexpression of connective tissue growth factor (CTGF). Inhibition of CTGF expression with siRNA is an attractive strategy to modulate the fibrotic mechanism, which could inhibit or reverse the process of fibrosis.^159^, ^160^

#### 3.3.1 Mesoporous silica nanoparticles

Recently, Kang et al.^161^ investigated the anti‐fibrotic activity of siRNA against CTGF using mesoporous silica nanoparticles (MSNs). MSNs are attractive carriers due to their high surface area and large pore sizes.^162^, ^163^ Moreover, the ease of surface modification makes them a suitable option for siRNA loading.^162^ One system named DegradaBALL (LEM‐S401) showed convenient loading, effective intracellular uptake, biocompatibility, biodegradability and sustained release of siRNA for durable CTGF gene‐silencing in TGF‐β‐induced skin fibrosis model cells. It also induced a knockdown of collagen types I and III, which are excess extracellular matrix components in fibrotic skin. LEM‐S401 also inhibited the formation of hypertrophic scars in wound‐associated dermal fibrosis mouse models, during both the epidermis recovery and tissue remodeling process. Notably, the authors also reported that with LEM‐S401, 10–1000 times less siRNA was sufficient to still induce more significant and prolonged gene knockdown compared to other siRNA drug candidates against skin fibrosis. Since silica (the base component of MSNs) is generally recognized as safe (GRAS) by the FDA, this class of materials has a high translatability to treat human hypertrophic scars and keloids.

### 3.4 Wound healing and scar therapy

Chronic wound healing remains a serious problem worldwide, causing a significant public health burden.^164^ This process is characterized by three stages: inflammation, proliferation, and remodeling. Aberrant wound healing can occur when any of these processes do not occur normally.^165^ The development of drug delivery strategies to the wound site is complicated by common features associated with aberrant wound healing, including poor vascularization and hypoxia, infection, oxidative stress, excess proteases leading to therapeutic degradation, and frequent mechanical disturbances during body movement.^166^, ^167^ These challenges necessitate the development of new topical wound healing therapies with improved efficacy.

Therapeutic strategies for wound healing include small molecule drugs, growth factors, stem cells, fibroblasts and RNA interference (RNAi) therapies such as siRNA and microRNA (miRNA).^167^ Among these, RNAi therapies provide an attractive option over as they are more targeted toward abnormal cells, providing a safer option to correct aberrant wound healing.^168^ These therapeutic drugs can often target genetic defects that lead to chronic wounds, for exaMPLE, certain genes overexpressed in diabetic patients.^169^ However, many RNAi therapies rely on scaffold‐based strategies, which are often delivered subcutaneously, an invasive method compared to passive topical delivery strategies.^170^, ^171^

#### 3.4.1 Lipid nanocarriers

While cationic lipids are widely used as topical siRNA delivery carriers,^172^, ^173^ neutral lipids and hybrid systems (e.g., PEG‐coated lipid nanoparticles) have been investigated to circumvent the side effects associated with cationic lipid systems.^174^ Recently, Rabbani and coworkers^175^ developed a hybrid lipoplex consisting of a DOTAP and sodium cholate (NaChol) to form DOTAP‐NaChol (DNC) LNPs combined with a cationic supercharged coiled coil protein (CSP) to enhance Keap1 siRNA delivery for treating diabetic wounds. The lipoproteoplex was complexed with siRNA to form stable particles that successfully delivered siRNA targeted against Keap1, when applied topically in a humanized murine diabetic wound healing model. This finding indicates that coiled‐coil proteins to enhance siRNA complexation and packaging within LNPs is a promising strategy for enhancing topical RNAi delivery.

#### 3.4.2 Inorganic nanoparticles

Inorganic nanoparticles, especially gold nanoparticles, are a popular nanocarrier for NA delivery due to their low toxicity and immunogenicity combined with ease of surface functionalization.^166^ For gold‐nanoparticle formulations, tightly packed siRNA is conjugated to the surfaces of the nanoparticles, resulting in SNA constructs. They display desirable properties such as nuclease resistance and the ability to enter cells via scavenger‐mediated endocytosis.^176^ Recently, the efficacy of SNA‐gold nanoparticle conjugates as a topical RNAi therapy was demonstrated by Randeria et al.^177^ who used this system for ganglioside‐monosialic acid 3 synthase GM3S knockdown in diabetic mice to enhance wound healing. When administered topically, the SNA nanoparticles decreased local GM3S expression by more than 80% and led to a twofold increase in the rate of wound healing with no obvious signs of toxicity. This is one of the first examples of SNA‐gold nanoparticles being used for topical delivery in wound healing, paving the way for future applications.

#### 3.4.3 Polymeric nanoparticles

Polymeric nanoparticles offer tunability combined with biocompatibility and protection of cargo degradation. Their charge and size can be optimized for use as oligonucleotide vehicles.^178^ PEI is a commonly used cationic polymer for siRNA delivery; however, associated cytotoxicity and non‐biodegradability limits its use.^178^, ^179^ Co‐polymerization of PEI with other polymers can mediate this issue, a strategy recently used by Cho and coworkers.^112^ The authors developed a degradable poly(sorbitol‐co‐PEI), a copolymer of sorbitol diacrylate with PEI, to deliver siRNA targeting CTGF in a cutaneous murine wound healing model. The delivery of siRNA using this system showed a significant reduction in scar contraction post‐healing with almost no contraction observed in some mice. Besides PEI, cyclodextrins are a class of natural oligosaccharides that are used for oligonucleotide delivery.^178^ They are generally nontoxic and non‐immunogenic, hence conjugation with PEI has been explored to improve biocompatibility. Li et al.^180^ reported the use of a cationic star‐shaped polymer having a β‐cyclodextrin core and PEI dendron arms to deliver matrix metalloproteinase MMP9 siRNA to reduce expression in skin fibroblasts and accelerate wound healing in diabetic rats. The polymer achieved a 1.5‐fold greater delivery than lipofectamine in vitro and significantly enhanced wound closure in vivo compared to free siRNA.

The introduction of an external stimuli‐responsive moiety in these polymeric carriers can enable temporal control of siRNA and miRNA release. Blersch et al.^181^ recently synthesized a library of light‐responsive polymeric nanoparticles to enable temporal control of intracellular release of noncoding RNAs. The authors incorporated a photocleavable o‐nitrobenzyl linker in copolymers of amine and bisacrylamide. One of the top candidates for in vitro transfection efficiencies, P1C7, was used to demonstrate light‐activated delivery of miRNA150 to an acute wound healing model in vivo. Significantly higher transfection efficiencies and wound closure rates were observed in groups treated with light and P1C7 compared to those treated with polymer alone or lipofectamine. While recent, this study lays the groundwork for the development of new polymeric nanocarriers responsive to light and other stimuli.

All of the aforementioned studies use an aqueous carrier solution for topical delivery. Particularly for wound healing, this is insufficient from a translational perspective. The mechanical stresses endured by wounds demand a more robust formulation accompanied by a protective dressing. Moving forward, RNAi‐based carriers for wounds should focus on formulation and application strategies to render these strategies more favorable for clinical translation.

#### 3.4.4 Layer‐by‐layer assemblies

Hammond et al.^182^, ^183^ used layer‐by‐layer (LbL) approaches to assemble ultrathin polymer films for localized and controlled delivery of siRNA to chronic wounds to accelerate wound healing in vivo. In their reports, a commercially available woven nylon bandage was applied directly into healing wounds for the delivery of siRNA.

**FIGURE 6:**
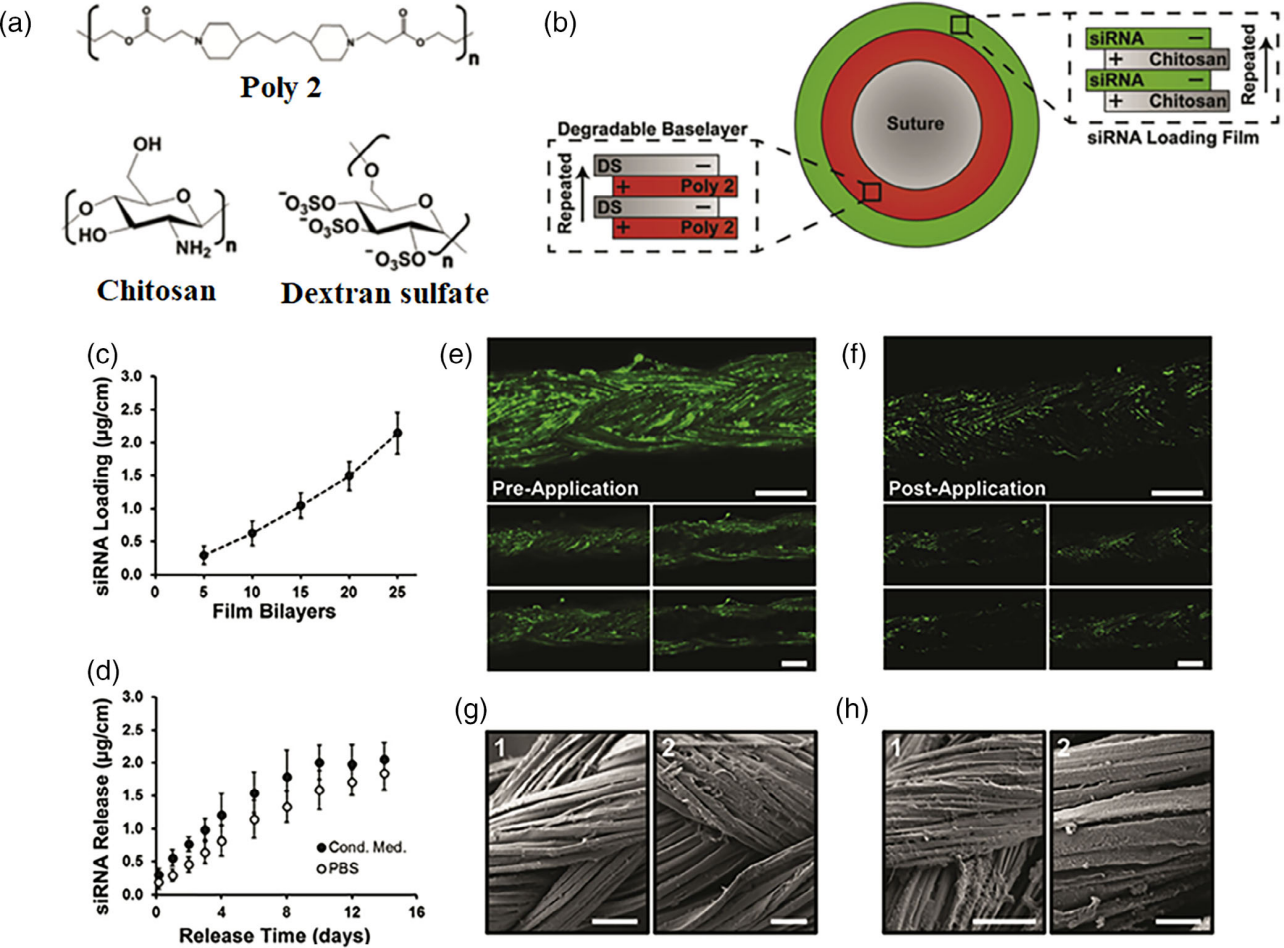
LbL film architecture applied to a commercially available silk suture for the controlled local delivery of siRNA. (a) Chemical structure of repeating unit of polymers used in film assembly. (b) Schematic illustration of the LbL assembly constructed on the suture surface. (c) siRNA incorporation per cm of coated suture at 5, 10, 15, 20, and 25 architecture repeats. (d) Release of siRNA over 14 days in vitro in either PBS or cell‐conditioned media at 37°C. (e) Confocal imaging of fluorescently labeled siRNA incorporated within film coating prior to degradation, and (f) 5 days into degradation in cell‐conditioned media. (g) SEM images of LbL‐coated silk sutures prior to degradation and (h) after 5 days in cell‐conditioned media. Reprinted (adapted) with permission^111^ from Copyright (2016) Elsevier Ltd

LbL assemblies have also been used for scar therapy. The process of wound healing often results in thick, collagen‐enriched tissue called scar tissue, which can negatively impact quality of life.^156^, ^157^ Cutaneous scars from serious traumatic injury can cause long‐lasting complications due to scar contraction and poor tissue remodeling reducing the range of motion and joint mobility and subsequently impairing function.^158^, ^184^ Castleberry et al.^111^ applied the LbL technology to anti‐scar therapy. The authors implemented the nanolayered platform to deliver anti‐CTGF siRNA and investigated its potential to improve scar outcomes in a third‐degree burn‐induced scar model in rats. CTGF is a therapeutic target within wounds to ameliorate fibrosis without impairing normal wound healing. They described an ultrathin polymer coating that can be uniformly assembled onto a commercially available suture using LbL assembly (Figure 6). Local application of this platform offered sustained knockdown of CTGF, resulting in significant alteration of the local expression of alpha‐smooth muscle actin, tissue inhibitor of metalloproteinase‐1, and collagen, which play important roles in scar formation. Knockdown of CTGF within the healing burn wounds showed improved tissue remodeling, reduced scar contraction and the regeneration of papillary structures within the healing tissue. The controlled local delivery of siRNA from ultrathin polymer coatings can be of potential benefit also for improved local cancer therapies as well.

#### 3.4.5 Alternative strategies for wound healing

Apart from RNAi, other biological therapeutics such as cytokines, cells and bacteria have been delivered topically to enhance wound healing. He et al. demonstrated the efficacy of topically delivered IL‐33 in accelerating wound closure.^185^ This was mainly attributed to polarization of wound‐associated macrophages to the pro‐healing M2‐like phenotype. Skin grafts generated in vitro are extensively used to treat wounds and burn injuries covering large areas. Regeneration of the dermis in vivo through delivery of fibroblasts is a robust strategy to replace skin grafts due to superior integration into the skin. Fibroblasts are often delivered in biodegradable scaffolds for local retention and survival, whereby the scaffold is gradually replaced by regenerated tissue. Recently, Loh and coworkers delivered fibroblasts to a wound site via nonbiodegradable scaffold made from bacterial cellulose and acrylic acid.^186^ Interestingly, delivery of fibroblasts alone did not significantly accelerate wound closure; however, co‐delivery with growth factors resulted in significantly faster healing. While nonbiodegradable scaffolds may be useful in situations where healing rates are slow, long‐term studies are needed to evaluate their safety. In a novel study, genetically modified *Lactobacilli* were delivered topically to a wound site, resulting in accelerated wound healing mediated by C‐X‐C motif chemokine 12 (CXCL12) production from the bacteria.^187^ This strategy provides an alternative to local protein delivery, which is limited by protein degradation. It is also safer than using genetically modified cells, such as mesenchymal stem cells, as probiotic bacteria are naturally present on skin.

### 3.5 Skin cancer

Cutaneous malignancies such as melanoma and nonmelanoma skin cancers represent a significant burden on global health.^163^ However, skin cancers frequently show blood vessel imperfections, which can facilitate uptake of nanocarriers administered topically. Despite this, elevated interstitial fluid pressure and excessively rigid extracellular matrix in the tumor environment pose an elevated challenge to transdermal delivery.^163^ Further, most therapies for skin cancer need to permeate deep into the basal epidermis or upper dermis to effectively act on relevant cell populations such as melanocytes.^188^ siRNA is an attractive therapy for skin cancer as it can be used to silence aberrant genes in cancer cells while achieving lower toxicity and improved patient compliance.^189^, ^190^, ^191^

#### 3.5.1 Lipid‐based nanocarriers

The favorable penetration of lipid‐based nanocarriers into the skin and their ability to effectively deliver NAs to the endosome has encouraged their use in treating cutaneous malignancies. Dorrani et al.^188^ reported the superiority of liposomes with NaChol edge activators in delivering BRAF‐silencing siRNA to human melanoma cells, resulting in more effective gene knockdown together with their ability to penetrate the basal epidermal layer of skin where melanocytes reside.

#### 3.5.2 Skin and cell‐penetrating peptides

Skin and cell‐penetrating peptides (SCPs) are short peptides with 5–30 amino acid residues that can target cell surfaces specifically. They can be screened for their affinity to certain receptors on the cell surface and subsequently used for targeted delivery.^23^, ^192^, ^193^, ^194^, ^195^ For example, Gan et al.^193^ used a screening process to identify an epidermal growth factor (EGF) receptor (EGFr)‐targeting peptide to target A431 squamous cell carcinoma. Ruan et al.^196^ combined SPACE with EGF to create a fusion protein that could specifically target melanoma cells that overexpress EGFr. The authors used this fusion protein carrier to deliver c‐Myc siRNA to a B16F10 mouse melanoma model in vivo, which significantly reduced tumor growth compared to naked siRNA. Notably, the effect the topically administered treatment was similar to that of systemically administered fusion protein siRNA.

SCPs have also been combined with polymers to form targeted nanocarriers. Wang et al.^109^ conjugated SCPs to HA and poly(β‐amino esters) (PBAE) to create a spontaneous micelle‐forming constructs (SHPs) having the ability to encapsulate siRNA and penetrate the SC. More importantly, the polymers showed pH responsive release with the fastest release occurring at the endosomal pH of 5.8, demonstrating specificity and reducing off target toxicity. The polymer‐siRNA construct was able to effectively deliver siRNA for survivin silencing in a B16F10 mouse melanoma model.

**FIGURE 7:**
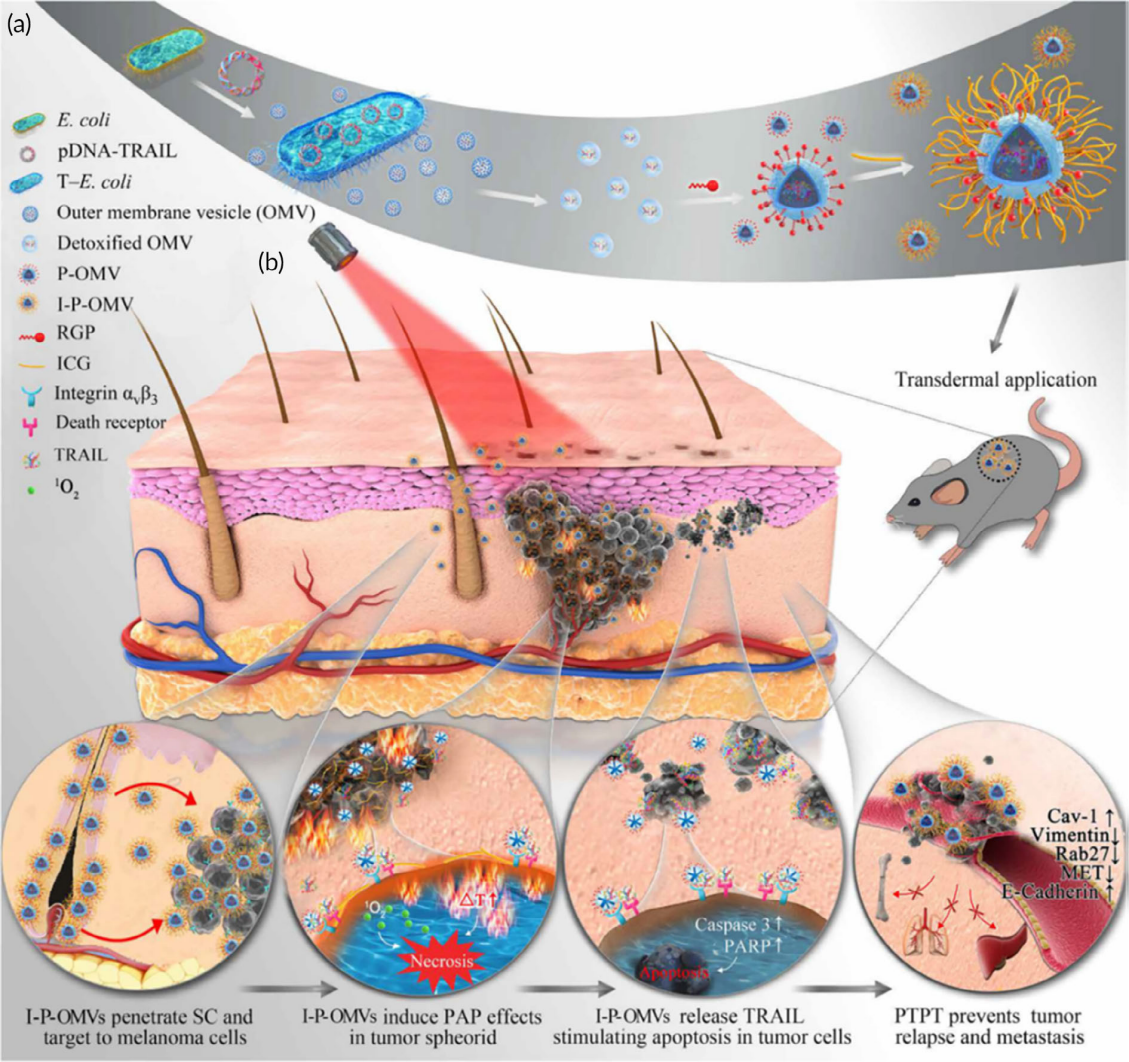
Schematic design and strategy for dermal photo‐TRAIL therapy against melanoma (I‐P‐OMVs with near‐infrared [NIR] light). (a) Preparation of I‐P‐OMVs: (i) transformation of *Escherichia coli* with pDNA‐TRAIL (T‐*E. coli*); (ii) isolation of OMVs from T‐*E. coli*; (iii) detoxification of OMVs with lysozymes; (iv) modification of OMVs with RGP‐forming P‐OMVs; and (v) conjugation of ICG to P‐OMVs forming I‐P‐OMVs. (b) Topical application of I‐P‐OMVs induces photo‐TRAIL treatment in skin melanoma: (i) I‐P‐OMVs penetrate through skin and target to melanoma; (ii) NIR irritation triggers ICG to induce hyperthermia effect and secret singlet oxygen that clears primary melanoma spheroids promptly; (iii) photothermal effect induces the deformation of OMVs that release TRAIL, followed by their binding to death receptors on surfaces of melanoma cells, activating the apoptosis in residual melanoma cells; and (iv) I‐P‐OMVs+NIR treatments prevent the metastatic potential of melanoma by interfering with relevant protein expression. Reprinted (adapted) with permission from^197^ under CC 4.0

Recently, tumor‐targeting peptides have been combined with bacterial‐outer membrane vesicles (OMVs) derived from transgenic *Escherichia coli* to topically deliver indocyanine green (I‐P‐OMVs) for a combination photo‐TNF‐related apoptosis‐inducing ligand (TRAIL) therapy against melanoma.^197^ The natural skin penetrating abilities of the OMVs were enhanced by incorporation of the a_v_b_3_ integrin‐targeting peptide to specifically target melanoma cells. The combination of TRAIL and phototherapy showed significantly higher in vivo efficacy compared to individual therapy and combination therapy without the OMV carrier (Figure 7).

#### 3.5.3 Mesoporous silica nanoparticles

**FIGURE 8:**
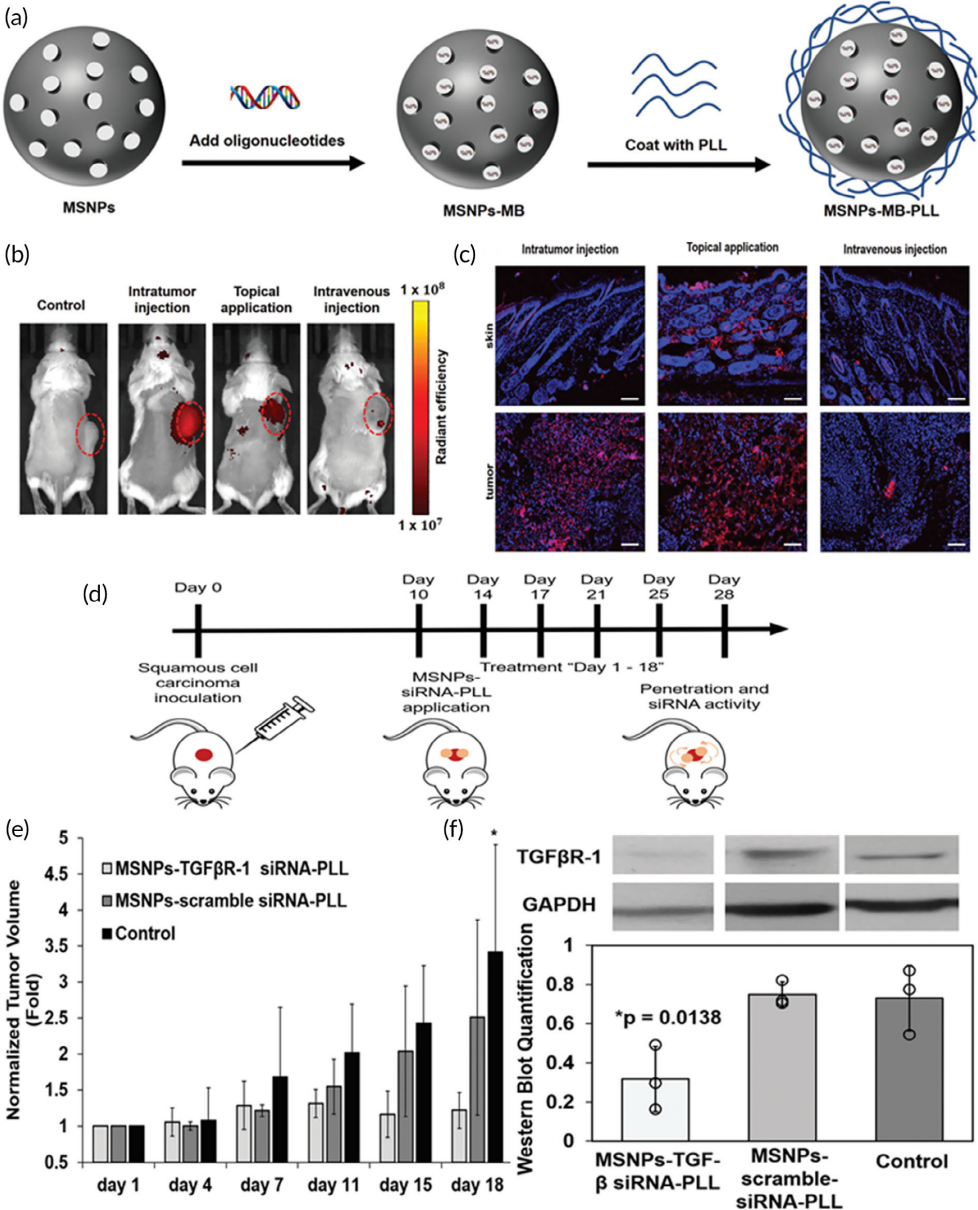
Topical delivery of siRNAs with MSNs for treating skin cancer. (A) Illustration of the synthesis of MSNP‐oligonucleotide complexes. (B) Representative in vivo imaging system (IVIS) images showing the biodistribution of Cy5 delivered using MSNs‐Cy5‐PLL administered to a mouse squamous cell carcinoma (SCC) xenograft model by topical, intratumoral and intravenous delivery. (C) Fluorescence images of the skin and tumor tissues. (D) In vivo efficacy of topical treatment in SCC model showing normalized tumor volume against that at Day 1. (E) Expression of TGFβR‐1 in the tumor tissue after treatment. Reproduced from with permission from^198^ The Royal Society of Chemistry

The effectiveness of MSNs for transdermal siRNA delivery to cutaneous cancer was recently demonstrated by Lio et al.^198^ who used MSNs to deliver TGFβR‐1 siRNA for treating squamous cell carcinoma in a mouse xenograft model. Notably, the MSNs‐siRNA constructs were surface‐coated with poly‐l‐lysine (PLL) to render a net cationic charge. Topical administration had sustained siRNA concentration within the tumor compared to intravenous or intratumoral administration and proved efficacy in suppressing tumor growth (Figure 8).

## 4 CRITICAL ANALYSIS AND OUTLOOK

Noninvasive dermal delivery systems allow simple application to different areas of the body in a patient‐friendly way. Transdermal patches are an ideal drug delivery platform that have been widely used in the clinic for the delivery of hormones and small‐molecule drugs; however, their use for vaccines and NA delivery is limited by poor permeation of biomacromolecules, limiting the amount of the cargo that can be delivered to the skin.^35^, ^199^ The efficiency of this technology can be improved when coupled with other tailored approaches that have shown success in dermal delivery. Such delivery systems can also be formulated in semi‐solid dosage forms as creams or gels for convenient topical application.

Novel transport‐promoting peptides can be identified via high‐throughput screening approaches or computational approaches as molecular docking to aid the design of peptides of improved stability. Also, using a combination of peptides with different functions can allow for synergistic effects by promoting skin and cellular penetration simultaneously.^142^, ^194^

Formulation approaches can be leveraged to formulate lipid or polymer‐based nanocarriers with excipients that possess multiple functionalities such as enhancing skin permeation, facilitating cellular uptake and enhancing the stability of the biological molecules either during storage or after administration until reaching the target cells.

Lipid‐based nanocarriers have become a clinically validated platform technology for delivering NAs of variable size, including saRNAs encoding certain components of SARS‐CoV‐2. Large‐scale manufacturing is feasible implementing rapid mixing and microfluidic procedures. This can be extended for delivering biomacromolecules into the skin in clinical practice. However, despite the promising outcomes shown by cationic lipids (e.g., DOTAP) in liposomes for complexing NAs, the clinical translation of such systems has been limited due to carrier‐related toxicity arising from the highly positive charges. In some cases, such lipids can inhibit the gene silencing process.^200^ Recently, a zwitterionic lipid (cephalin) was found to be more effective than DOTAP in delivering saRNA to human skin explants.^200^ Ionizable cationic lipids that have near‐neutral charge at physiological pH and become charged in acidic compartments provide the advantage of being less toxic and more efficient in entrapping NAs while facilitating endosomal escape. Thus, a key advancement for effective LNC‐mediated dermal delivery of NAs will rely on screening lipid libraries and analyzing the structure–activity relationship for optimizing the carrier properties for cutaneous applications.^201^

For polymeric nanoparticles, PEI and its derivatives are the most commonly used polymers for treating skin diseases with NAs. To overcome PEI‐associated toxicity, the polymer can be copolymerized with more biocompatible moieties such as cyclodextrin^202^ or conjugated to other polymers such as PLGA.^203^ PBAE is another class of cationic polymers with a lower toxicity than PEI. PBAE nanoparticles have shown promise in delivering DNA in an epidermolysis bullosa model and have led to the first topical gene therapy product licensed by Amryt Pharma in 2018.^204^ Natural polymers like CS and HA provide biocompatible options for various polymer‐based nanocarriers and have shown promising outcomes for dermal delivery of biologics. Bioconjugation chemistry can be harnessed to design and optimize stimuli‐responsive conjugates that enable skin penetration and cargo release upon exposure to the target microenvironment.^2^ Further modification with targeting moieties can enable delivery to specific cell subtypes within the skin.

ILs are a relatively recently studied drug delivery system whose physicochemical properties can be tuned to enhance the transport and stability of biological molecules. They are designer materials that can be tailored to attain the desired concentration and adequate viscosity desired for topical application without the need for thickening agents. They can also be formulated with cation and anion counterparts that possess a desirable pharmacological effect, acting as bioactive delivery system to augment the efficacy of the loaded bioactive cargo. They offer a promising tool for noninvasive dermal delivery of antigens and NAs. Recently, the use of ILs has moved to into clinical trials. Ko and coworkers reported the clinical translation of CAGE for the treatment of rosacea, which is an inflammatory skin disease.^205^ Efforts to elucidate the mechanisms of transdermal transport, evaluate potential toxicity and describe interactions with different biological molecules can strengthen the transformative potential of other ILs and widen their clinical utility for the delivery of biologics to the skin.

## 5 STATE OF CLINICAL TRANSLATION

Dermal delivery of NAs can have a potential impact in the clinic due to the existence of many skin diseases with NA therapeutic targets accounting for their pathogenesis.^15^ Currently, clinical trials for NA therapies for cutaneous disorders are performed by intradermal injection. The first siRNA drug (TD101) against pachyonychia congenita was developed by Transderm Pharmaceuticals Inc., and it was found to be safe in Phase I results.^113^, ^206^ RXi Pharmaceuticals tested the siRNA drug RXI‐109 to suppress CTGF expression, based upon the sd‐rxRNA (self‐delivering RNAi compound) platform. Recently, Phase II results for RXI‐109 were announced, which showed the expected safety profile, prevented the formation of hypertrophic scars and reduced the reappearance of keloids.^207^, ^208^ Another siRNA drug that targets CTGF, OLX101, was designed by OLiX pharma for the treatment of hypertrophic scars and keloids. Intradermal injection achieved effective gene silencing in animal models and is currently under Phase I studies.^209^ A dual‐targeting siRNA, STP705, was designed by Sirnaomics, Inc. for managing fibrosis and inflammatory activity. It comprises a polypeptide nanoparticle for enhanced delivery of two siRNA targets: one for TGF‐β1 and the other targeting to cyclooxygenase‐2 (*COX2*) gene. This drug has proceeded to Phase II clinical trials in patients with hypertrophic scars.^210^, ^211^

Apart from intradermal injection, other current clinical trials are based on physical disruption methods (mainly microneedles and microjets) with exception for a few transdermal patches that are used for the dermal delivery of some peptide therapeutics. Helix BioPharma developed a cream containing IFNα‐2b encapsulated in liposomes that was used in Phase II human trials for treating low‐grade squamous intraepithelial lesions.^212^ Only one recent Phase II clinical trial was performed for the dermal delivery of an anti‐TNF antibody using a hydrogel formulation (NCT01936337). However, despite the wide research on using nanocarriers for NA delivery to the skin, most of the studies are still in an early preclinical phase. More effort should be directed to explore new transporter mechanisms and to identify synergistic combinations to overcome the skin barrier with minimal invasiveness or irritation. These efforts should be extended to deliver antibodies given their potential role in treating a variety of skin conditions.

It is worth pointing out that combining noninvasive active delivery techniques with the discussed formulation approaches can synergistically promote dermal delivery and achieve better therapeutic outcomes. While not discussed in detail in this review, iontophoresis and sonophoresis have shown promising outcomes in this regard. For example, iontophoresis has been employed to deliver cetuximab, an anti‐EGFR monoclonal antibody, to the deep layers of skin.^21^ Combining iontophoresis with cetuximab‐conjugated liposomes showed promising outcomes in a squamous cell carcinoma mouse model.^213^ This technique has also been used to deliver the anti‐TNF‐α drug etanercept for topical psoriasis.^214^ The combined use of PAMAM dendrimers as carriers for antisense oligonucleotides (ASOs) and iontophoresis caused more than a twofold reduction in tumor volume in a skin cancer model compared to iontophoresis alone or the passive delivery of a ASO‐dendrimer complex.^190^ The combined use of iontophoresis with charged liposomes has also been reported to enhance the TCI using OVA and silver nanoparticles.^215^

While sonophoresis has been reported to improve the delivery of a variety of macromolecules,^216^ few reports exists on its combined use with other carriers. Low frequency ultrasound has been implemented to enhance dermal penetration of quaternized starch and miR‐197 (a microRNA targeting subunits of IL‐17 and IL‐22 receptors) complexes. This showed a significant reduction in psoriatic levels when tested in a human skin xenograft model in mice.^107^ Sonophoresis also enhanced the permeation of PAMAM and low molecular weight peptide dendrimers, while increased the dermal retention of high molecular weight peptide dendrimers.^217^ Given the ability of dendrimers to form complexes with siRNA, sonophoresis may be useful to further enhance their dermal delivery.

Iontophoretic patches and sonophoretic devices can be used for self‐administration. However, use of such combinations should be carefully considered (e.g., the intensity of current or frequency of the ultrasound) to ensure that delivery is directed to target layers of skin while avoiding further penetration into systemic circulation. The combinatorial use of active techniques with rationally designed formulations may help the performance of therapeutic moieties at lower doses, while using physical methods at lower strengths to ensure safety and potentially enable a take‐home therapy.

## 6 REMAINING CHALLENGES

Despite the recent efforts to advance noninvasive dermal delivery of vaccines and topical NA therapies (summarized in Table 1), there still exist some limitations to overcome before these systems can become clinically viable.

**TABLE 1:** A summary of formulation‐based strategies for dermal delivery of antigens and nucleic acids

Delivery system	Composition	Cargo	Application(s)	REF
Peptides	TD‐1	GAPDH siRNA	Dermal RNAi delivery for gene silencing	132
	SPACE	IL‐10, GAPDH siRNA	Dermal RNAi delivery for gene silencing	133
	TAT peptide, Arginine‐rich peptide combined with TJ‐opening peptide (AT1002)	siRelA	Atopic dermatitis	124, 137
	Amphipathic peptide Mgpe9	Plasmid DNA	Dermal gene delivery	138
	SPACE‐EGF fusion protein	c‐Myc siRNA	Melanoma	196
Solid‐in‐oil systems	Cyclohexane, IPM, Sucrose laurate (L‐195)	β‐lactoglobulin, R‐848	Whey allergy vaccination	54
		T cell epitope peptides, pollen extract‐galactomannan conjugate	Pollinosis vaccination	50, 56
		OVA	E.G7‐OVA vaccination	58
	Cyclohexane, IPM, Sucrose laurate (L‐195), Sucrose erucate (ER‐290)	K‐TRP‐2 peptide, R‐848	Melanoma lung metastasis vaccine	57
Hyaluronic acid conjugates	HA‐dodecylamine‐OVA	OVA	EG7‐OVA vaccination	66
Lipid‐based nanocarriers	Liposome conjugated to LC‐targeting ligand	Doxorubicin, protein antigens	Langerhans cell histiocytosis	82, 84
	Transfersome	Hepatitis B surface antigen plasmid DNA	Vaccination against Hepatitis B	90
	Stearylamine ethosome	OVA	EG7‐OVA vaccination	89, 91
	Ethosome modified with galactosylated chitosan and coated with HA	OVA	EG7‐OVA vaccination	92
	Ultra‐deformable archaeosome	*Leishmania* antigen	Vaccination against *Leishmania*	97
	DSPC, CTAB, Cholesterol and Pyrrolidinium lipid	Erlotinib, IL‐36 siRNA	Psoriasis	198
	Liposome with NaChol edge activator	BRAF siRNA	Melanoma	174
Lipid‐peptide hybrids	Ethosome‐SPACE peptide	siRNA	Transcutaneous RNAi delivery	108
	Liposome‐AT1002 peptide	siRelA	Atopic dermatitis	109
	Lipoproteoplex with CSP	Keap1 siRNA	Diabetic wound healing	160
	*Escherichia coli* outer membrane vesicles with avb3 integrin targeting peptide	Indocyanine green for photo‐TRAIL therapy	Melanoma	197
Polymeric nanoparticles	Chitosan nanocapsule	OVA, vitamin E adjuvant	Transcutaneous antigen delivery	72
	HA nanocapsule	OVA, adjuvant	Transcutaneous antigen delivery	76, 78
	Mannosylated PEI copolymer	saRNA	Transcutaneous gene delivery	155
	Poly(sorbitol‐co‐PEI) copolymer	CTGF siRNA	Acute wound healing	112
	β‐cyclodextrin core with PEI dendron arms	MMP9 siRNA	Diabetic wound healing	180
	Light‐responsive bisacrylamide‐amine copolymer	miRNA150‐5p	Acute wound healing	181
Polymer‐peptide hybrids	HA‐PAE polymer‐conjugated cell‐penetrating peptide	Survivin siRNA	Melanoma	109
Polymer films and scaffolds	LbL ultrathin polymer film	CTGF siRNA	Chronic wound healing	183
	LbL ultrathin polymer film of chitosan and dextran sulfate	CTGF siRNA	Fibrosis amelioration	111
	Nonbiodegradable cellulose‐acrylic acid scaffold	Fibroblasts with growth factors	Wound healing skin graft	186
Virus‐like particles	Self‐assembled VLPs	HBV antigen, HIV‐1 precursor protein	Transcutaneous immunization	99, 101
Lipid‐polymer hybrids	Nanostructured lipid carrier core (glycerol stearate and oleic acid) coated with PEI	TNF‐α siRNA, tacrolimus	Psoriasis	141
Ionic liquids	Choline‐oleic acid	Water‐soluble antigen peptide	EG7‐OVA vaccination	106
	CAGE, CAPA	NFKBIZ siRNA	Psoriasis	103, 146
Liquid crystals	Monoolein, oleic acid, PEI Liquid crystal	IL‐6 siRNA	Psoriasis	149
Spherical nucleic acids	Cholesterol conjugated oligonucleotides in liposomes	IL‐17 siRNA	Psoriasis	150
	Thiolated SNAs/gold nanoparticles	EGFR siRNA	Psoriasis	151
	SNA‐gold nanoparticle conjugate	GM3 siRNA	Diabetic wound healing	177
Dendrimers	Cationic PAMAM dendriplex	TNF‐α siRNA	Psoriasis	153
	Orthinine‐derived dendrimers	saRNA	Psoriasis	110
Oil‐in‐water creams	IL‐23 aptamer	IL‐23 aptamer	Psoriasis	154
Mesoporous silica nanoparticles	DegradaBALL (LEM‐S401)	CTGF siRNA	TGF‐β induced skin fibrosis	161
	Poly‐L‐lysine‐coated MSN	TGFbR‐1 siRNA	Squamous cell carcinoma	198
STAR particles	Star‐shaped aluminum oxide particle	Tetanus toxoid vaccine	Transcutaneous immunization	102

The use of peptides and dendrimers may be constrained to some NAs with a certain size as SiRNA and oligonucleotides as opposed to DNA. Lipid nanoparticles and cationic polymers, despite their efficiency for cytosolic delivery, still suffer from some toxicity and instability issues. Chemical conjugations, despite being tunable to provide cellular targetability and controlled release rates, suffer from time‐consuming and labor‐intensive processes.

The stability of the biological macromolecules during manufacturing and storage is of critical importance. Care should be taken during the manufacturing process to avoid excessive agitation or shear, as well as drastic changes in pH, ionic strength, or temperature, as those factors can affect the molecular structure and result in a loss of biological activity.^218^, ^219^

When designing lipid or polymer‐based nanocarriers, the formulation parameters such as the choice of excipients and the loading method should be optimized based on the individual properties of the macromolecule and its possible interaction with any of the formulation ingredients. What works with a particular cargo might adversely affect the properties and transport of others.

Another important aspect is the translatability of the experimental results obtained with animal models to humans. With exception to studies performed on human skin explants, the majority of the studies have shown success in small animal models. One should take into consideration the biological and structural differences before extrapolating findings to humans since the human skin is thicker, has fewer hair follicles and is less permeable than rodent skin.

## 7 CONCLUSIONS

Looking at the clinical landscape for vaccines and cutaneous disorders reveals the critical need for noninvasive approaches to deliver biological macromolecules. Recent efforts have been made to overcome the barrier function of the skin by implementing a variety of smart delivery systems. To further advance this field, we need a better understanding and practice of how to deliver various macromolecules safely and efficiently to target cells. Translational value of preclinical work can be increased by using human‐relevant models such as human skin explants, tissue‐engineered skin models, and human‐based tissues grafted onto mice. Addressing these aspects and combining them with a deeper understanding of cell biology can lead to the improved design of bioengineered materials to navigate the skin and improve prophylactic and therapeutic outcomes.

## AUTHOR CONTRIBUTIONS

**Marwa Sallam:** Conceptualization; data curation; methodology; writing‐original draft. **Supriya Prakash:** Data curation; writing‐original draft. **Ninad Kumbhojkar:** Data curation; writing‐original draft. **Wyatt Shields:** Writing‐original draft. **Samir Mitragotri:** Conceptualization; writing‐review & editing.
